# Supplementary material for: Cellular reprogramming in vivo initiated by SOX4 pioneer factor activity
Source: Nat Commun. 2024 Feb 26;15:1761. doi: 10.1038/s41467-024-45939-z (PMC10897393; doi:10.1038/s41467-024-45939-z)
Supplement: Supplementary file 1 — Supplementary Information [file 41467_2024_45939_MOESM1_ESM.docx]

**Supplementary Information**

**Cellular reprogramming *in vivo* initiated by SOX4 pioneer factor activity**

Takeshi Katsuda^1,2,3^, Jonathan Sussman^1,2,3,4^, Kenji Ito^1,5^, Andrew Katznelson^1,5^, Salina Yuan^1,2,3^, Naomi Takenaka^1,5^, Jinyang Li^1,2,3^, Allyson J. Merrell^1,2,3^, Hector Cure^1,2,3^, Qinglan Li^1,5^, Reyaz Ur Rasool^1,3,6^, Irfan A. Asangani^1,3,6^, Kenneth S. Zaret^1,4,6*^, Ben Z. Stanger^1,2,3,5,*^

1. Perelman School of Medicine, University of Pennsylvania, Philadelphia, Pennsylvania.
2. Department of Cell and Developmental Biology, University of Pennsylvania, Philadelphia, PA
3. Abramson Family Cancer Research Institute, University of Pennsylvania, Philadelphia, PA
4. Graduate Group in Genomics and Computational Biology, Perelman School of Medicine, University of Pennsylvania, Philadelphia, PA
5. The Institute for Regenerative Medicine, University of Pennsylvania Philadelphia, PA
6. Penn Epigenetics Institute, University of Pennsylvania, Philadelphia, PA

**Content:**

**Supplementary Fig. 1.** Gating strategy for flow cytometry of DDC-induced reprogrammed cells and the representative results of each time point.

**Supplementary Fig. 2.** Gating strategy for FACS sorting of Rep_late and biliary epithelial cells.

**Supplementary Fig. 3.** Gating strategy for FACS sorting of Rep_early and Rep_intermed cells.

**Supplementary Fig. 4.** *Sox4* and *Sox9* are the most abundantly expressed *Sox* genes in the liver upon DDC-induced reprogramming.

**Supplementary Fig. 5.** Confirmation of SOX4 expression at the protein level.

**Supplementary Fig. 6.** Gating strategy for determination of reprogramming efficiency of hepatocytes expressing *Sox4*, *Sox9* or both.

**Supplementary Fig. 7.** Further characterization of Sox4- and Sox9-expressing hepatocytes.

**Supplementary Fig. 8.** Characterization of Sox4-expressing hepatocytes by RNA-Seq.

**Supplementary Fig. 9.** Kinetic analysis of phenotypic changes following Sox4 expression and the correlation heatmap of the ATAC-Seq data.

**Supplementary Fig. 10.** Representative browser views of ATAC-Seq data and profiling the Sox4 binding sites in hepatocytes by CUT&RUN-Seq.

**Supplementary Fig. 11.** Validation of consistency in CUT&RUN-seq data among three replicates and profiling the SOX4 binding sites in hepatocytes by CUT&RUN-Seq.

**Supplementary Fig. 12.** Gene ontology (GO) analysis for upregulated or downregulated genes during DDC-induced reprogramming.

**Supplementary Fig. 13.** Genomic distribution of Sox4-induced changes in chromatin accessibility and characterization of newly closed regions.

**Supplementary Fig. 14.** Quality control and quantification of purified recombinant MYC-FLAG-HNF4A and FLAG-SOX4 protein by SDS-PAGE using a BSA standard

**Supplementary Fig. 15.** Characterization of MORs and DORs in the ectopic Sox4 expression and DDC-induced reprogramming contexts.

**Table S1**. List of known hepatocyte and biliary/reprogrammed cell genes.

**Table S2.** PCR primers used for cloning by NEBuilder assembly.

**Table S3**. Antibodies used in this study.

**Table S4.** Primers used for qRT-PCR**.**

(**Supplementary Data 1-3** are provided separately as spreadsheets)


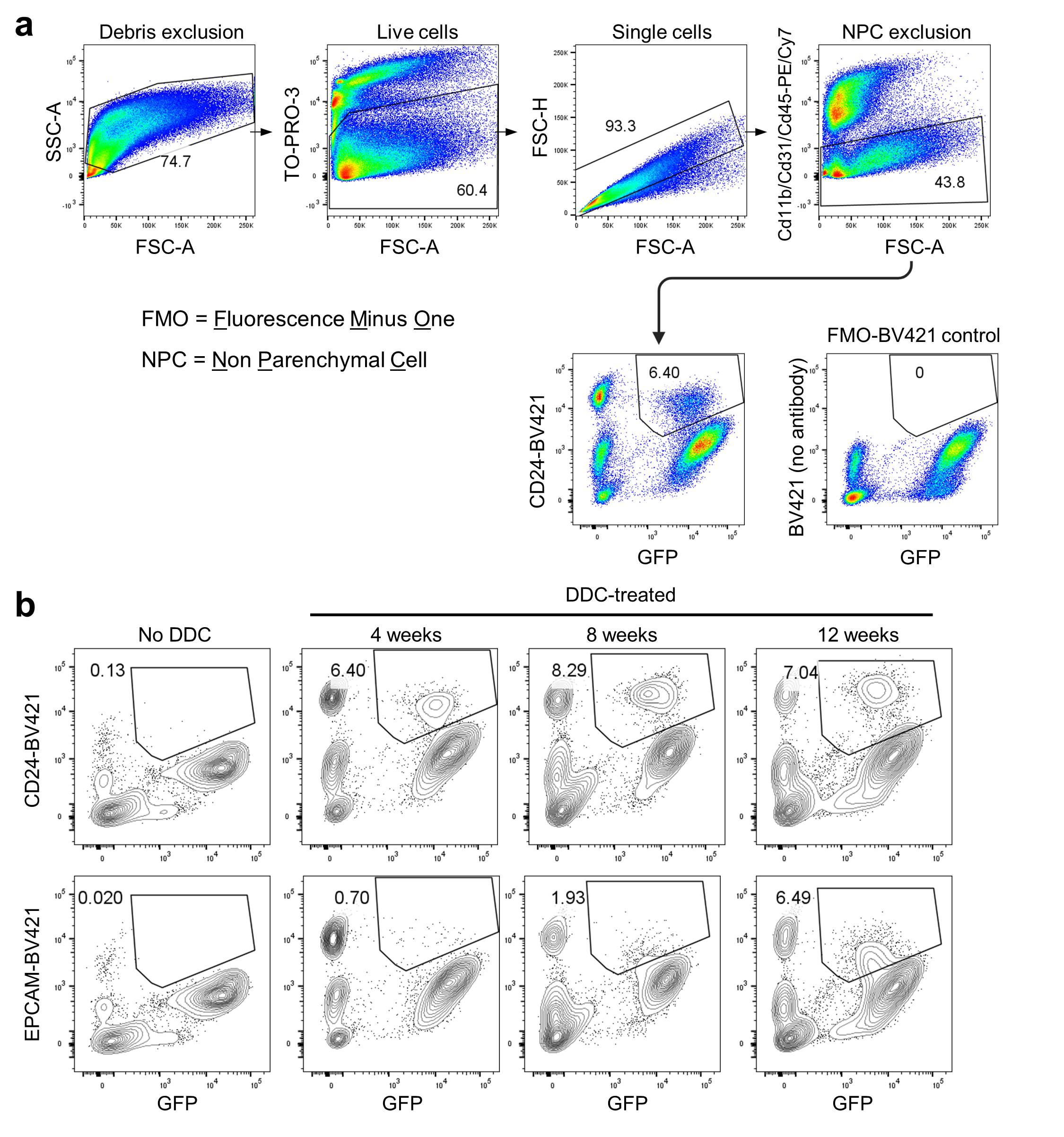


**Supplementary Fig. 1. Gating strategy for flow cytometry of DDC-induced reprogrammed cells and the representative results of each time point.**

1. Representative plots of gating strategy for flow cytometry of whole liver cells challenged with DDC for the designated time points (**Fig. 1a**). Cells harvested from normal livers (No DDC) serve as the week 0 control.
2. Representative plots indicating the reprogrammed cell fractions at the designated time points. The rectangle gates indicate the GFP/BV421 double positive cells with percentage of the total parental population. The percentages shown in **Fig. 1a** are calculated as (GFP+/BV421+)/(GFP+) ratios.


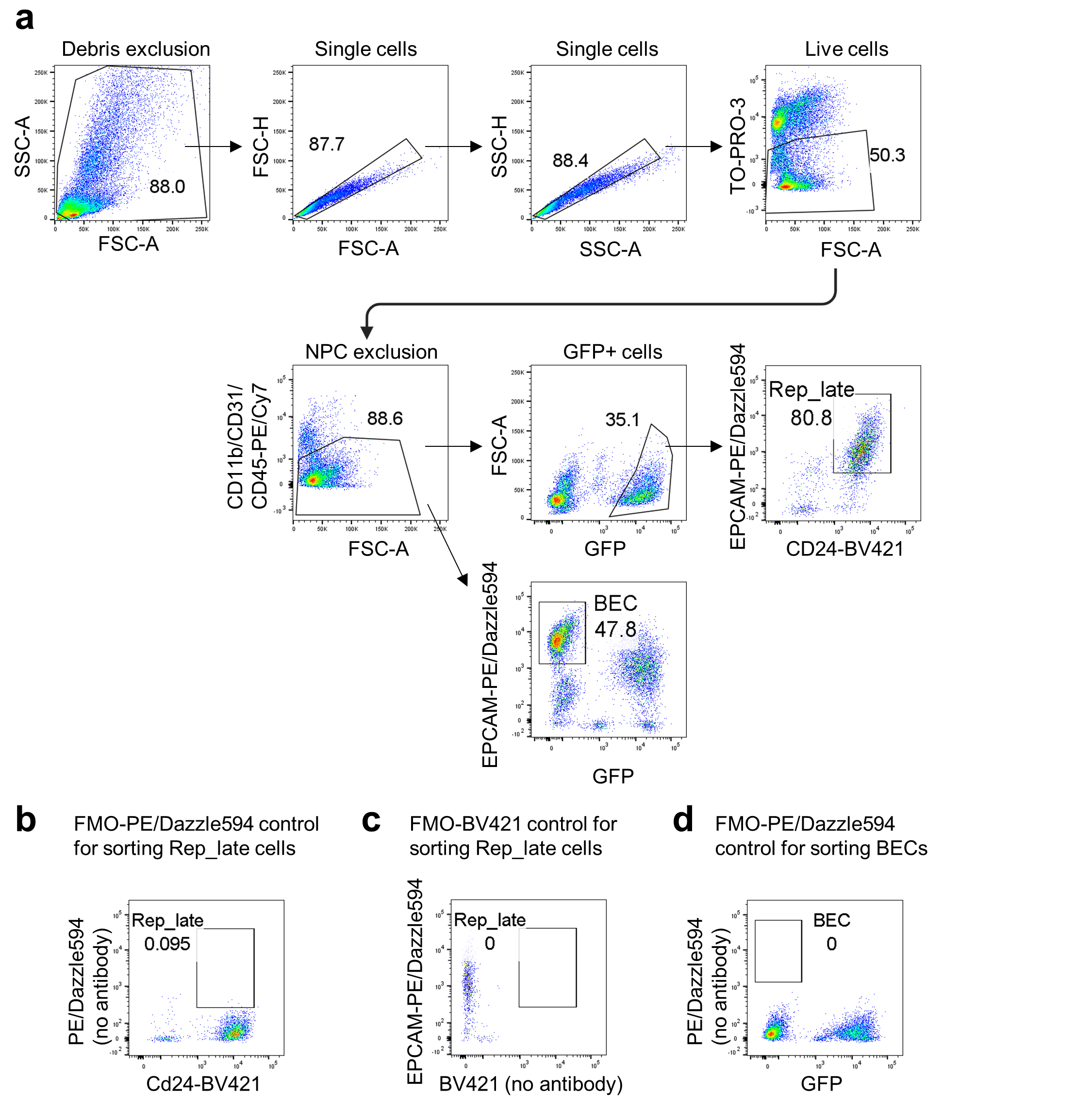


**Supplementary Fig. 2. Gating strategy for FACS sorting of Rep_late and biliary epithelial cells.**

1. Representative plots of gating strategy for FACS sorting of Rep_late and biliary epithelial cells using EPCAM+ fraction which was enriched by MACS (**Fig. 1b**).
2. Representative plots of PE/Dazzle594-fluorescence minus one (FMO) control, which was used to determine the threshold of Epcam expression of Rep_late cells.
3. Representative plots of BV421-FMO control, which was used to determine the threshold of CD24 expression of Rep_late cells.
4. Representative plots of PE/Dazzle594-FMO control, which was used to determine the threshold of CD24 expression of biliary epithelial cells.


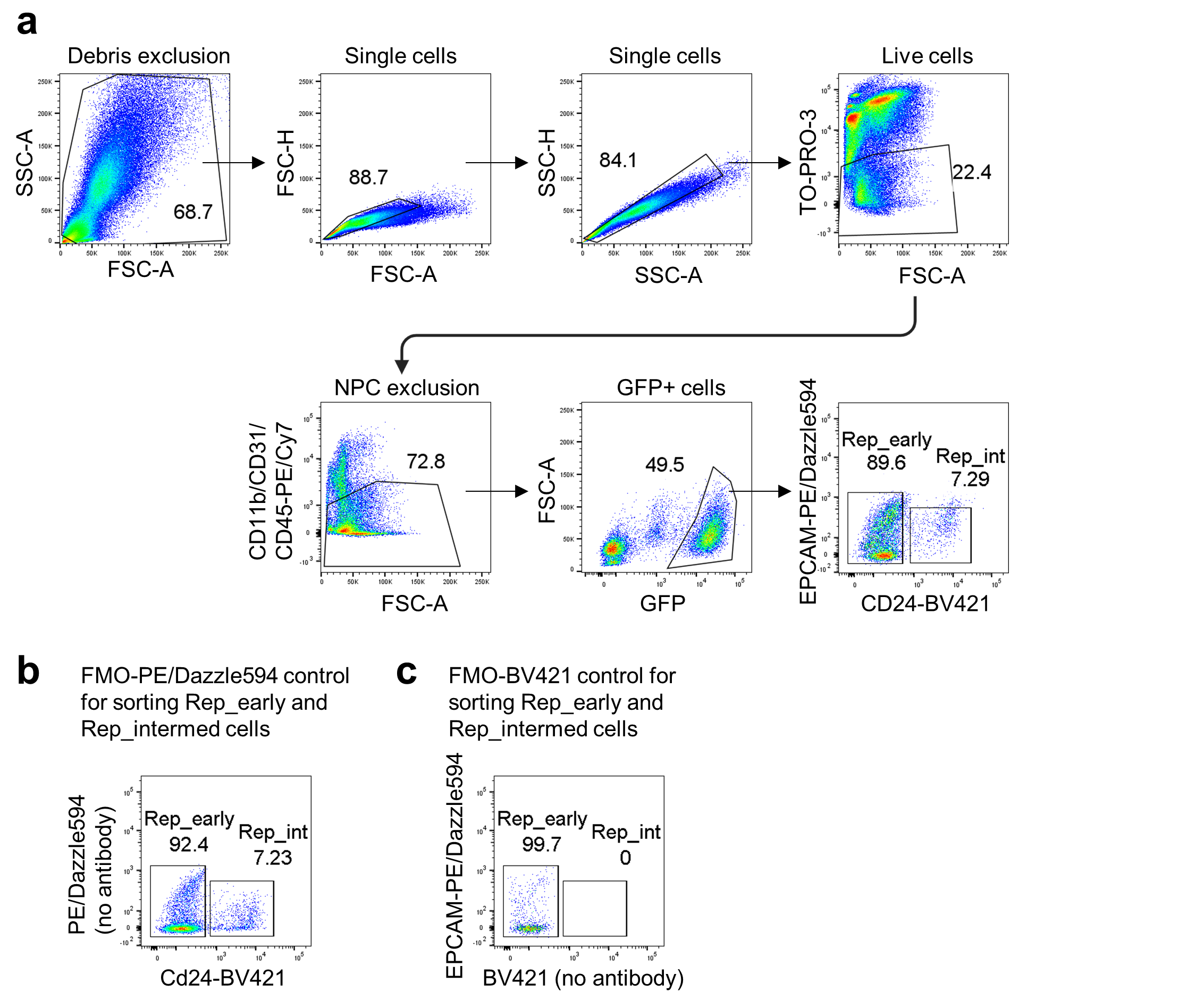


**Supplementary Fig. 3. Gating strategy for FACS sorting of Rep_early and Rep_intermed cells.**

1. Representative plots of gating strategy for FACS sorting of Rep_early and Rep_intermed cells using EPCAM- fraction which was enriched by MACS (**Fig. 1b**).
2. Representative plots of PE/Dazzle594-FMO control, which was used to determine the threshold of EPCAM expression of Rep_early and Rep_intermed cells.
3. Representative plots of BV421-FMO control, which was used to determine the threshold of CD24 expression of Rep_early and Rep_intermed cells.


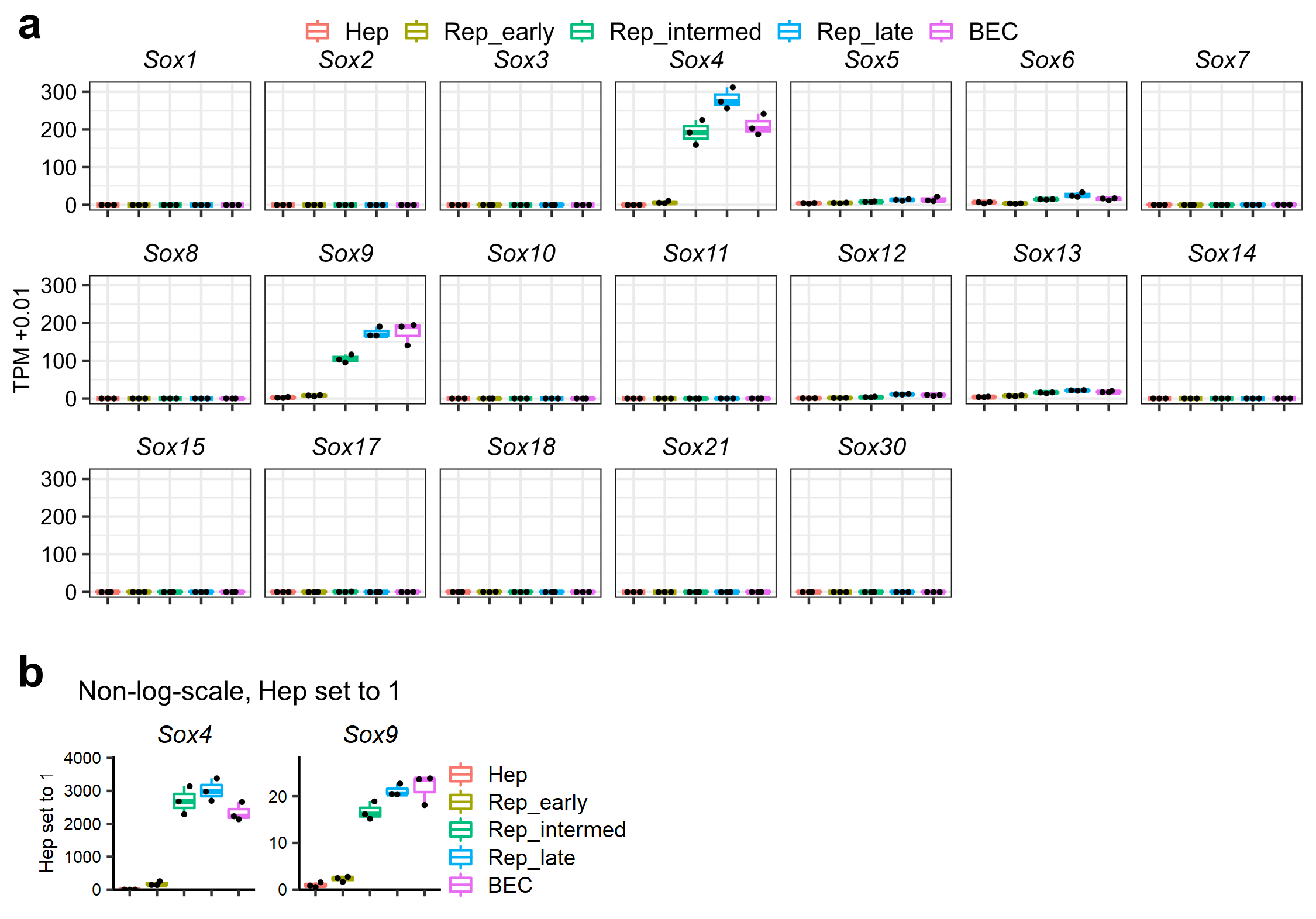


**Supplementary Fig. 4. *Sox4* and *Sox9* are the most abundantly expressed *Sox* genes in the liver upon DDC-induced reprogramming.**

1. Expression profiles for all Sox genes as quantified by RNA-Seq, normalized by transcripts per million (TPM) (n = 3).
2. Data for *Sox4* and *Sox9* with the median-based normalization method to illustrate differences compared to hepatocytes (Hep) as the baseline (n = 3).


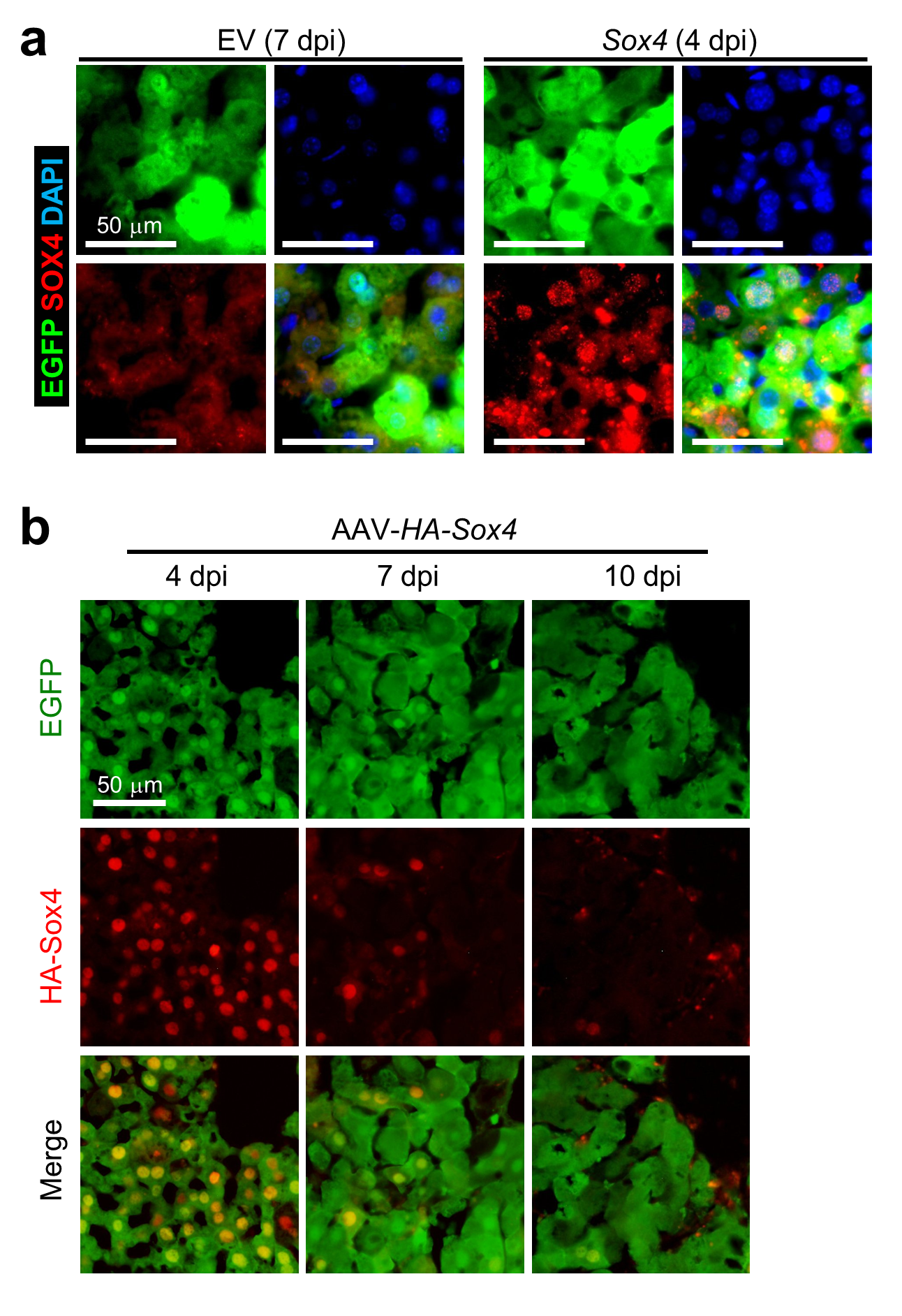


**Supplementary Fig. 5. Confirmation of SOX4 expression at the protein level.**

1. Individual images of each channel for SOX4 IF in **Fig. 2c**.
2. Kinetics of Sox4 protein expression as assessed by immunofluorescence using an anti-HA-tag antibody. Scale bar = 50 μm.


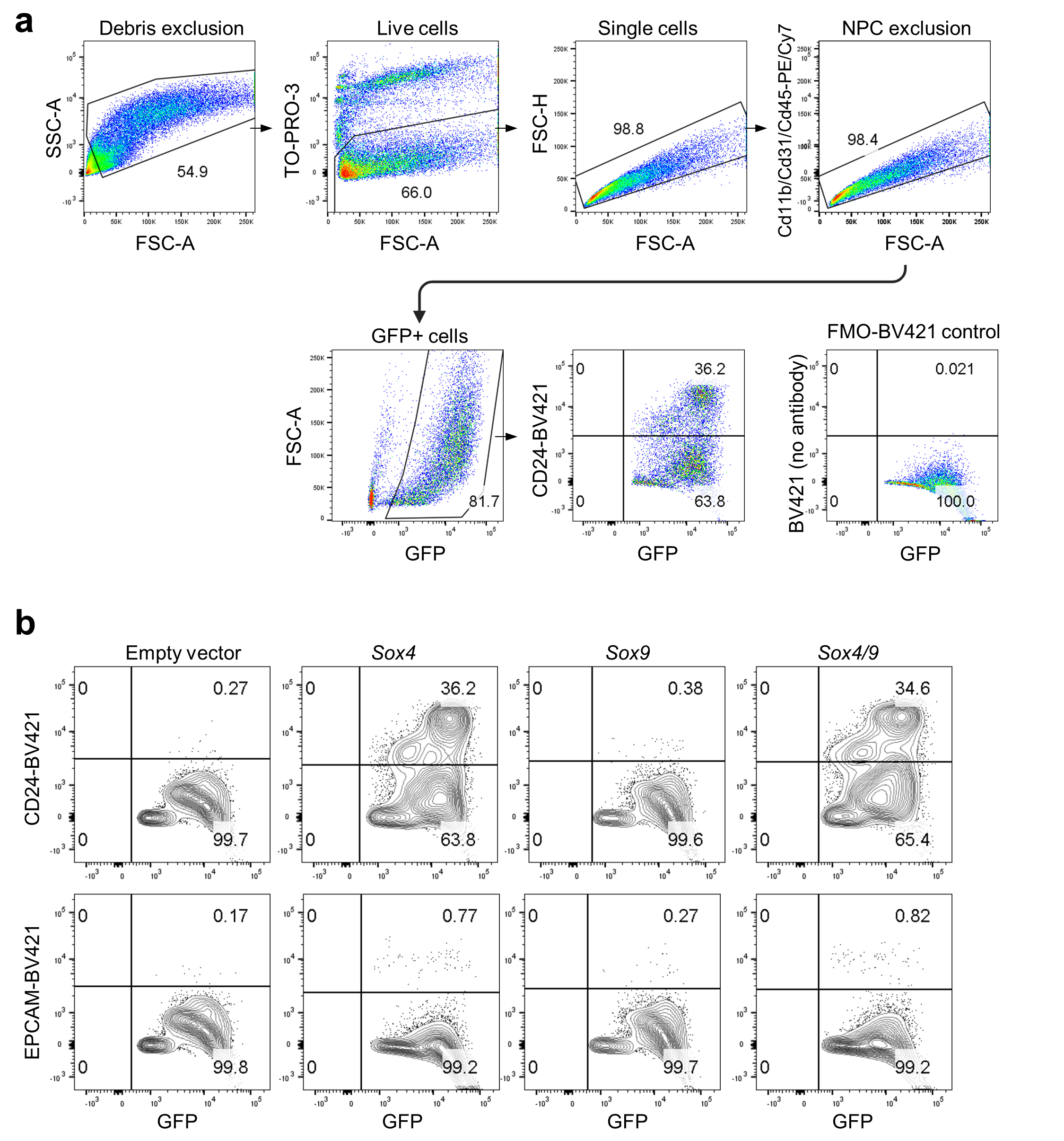


**Supplementary Fig. 6. Gating strategy for determination of reprogramming efficiency of hepatocytes expressing *Sox4*, *Sox9* or both.**

1. Representative plots of gating strategy for flow cytometry of whole liver cells expressing *Sox4*, *Sox9* or both at 7 dpi (experimental design described in **Fig. 2a**). Empty vector-injected cells serve as a control.
2. Representative plots indicating the reprogrammed cell fractions in the early (CD24) and late (EPCAM) stages. The percentages shown in the right top quadrant indicates GFP/BV421 double positive ratios based on the total GFP positive cells.


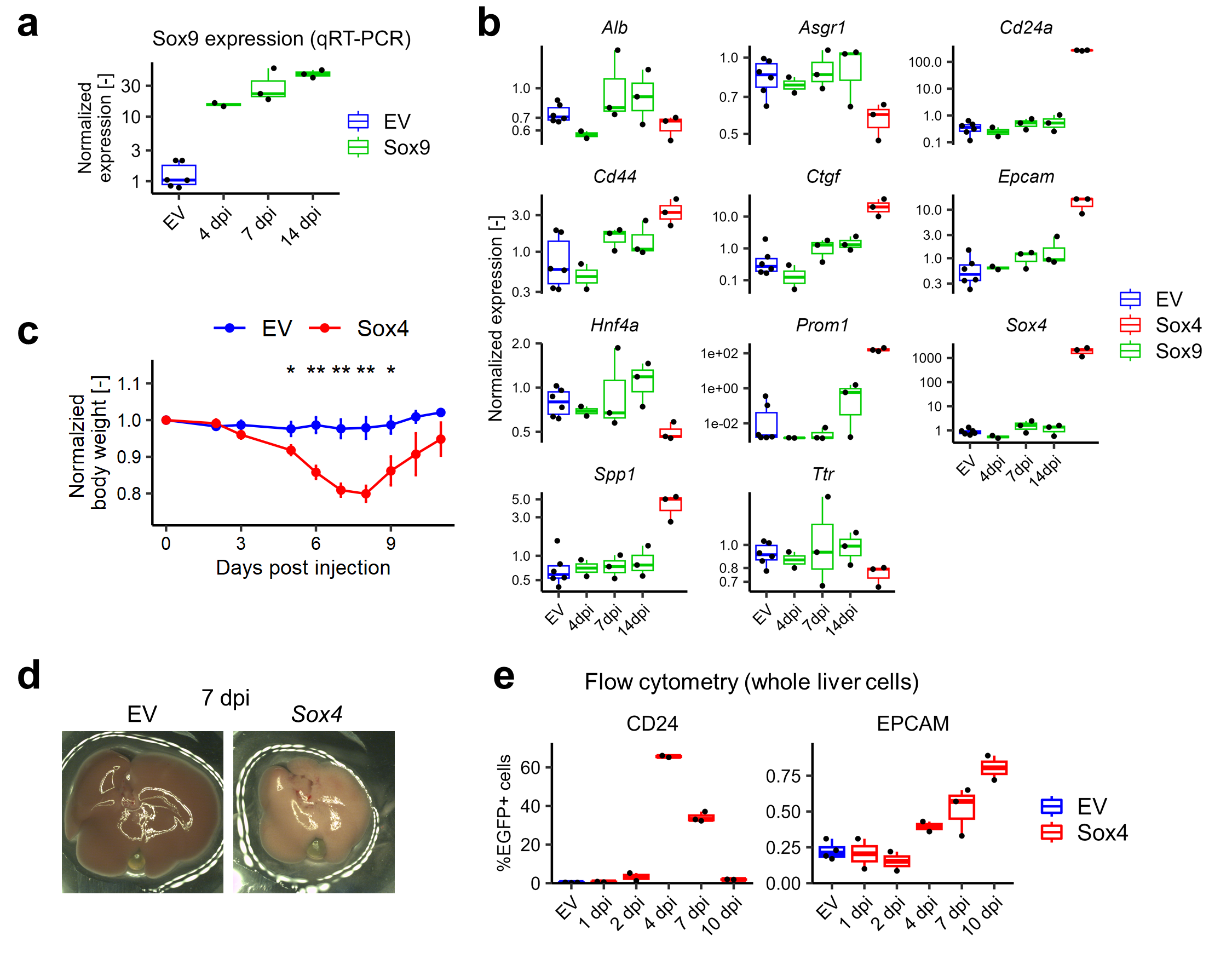


**Supplementary Fig. 7. Further characterization of *Sox4*- and *Sox9*-expressing hepatocytes.**

1. Time course of *Sox9* expression as assessed by qRT-PCR following ectopic *Sox9* expression in hepatocytes (n = 2-3).
2. Time course of expression changes in hepatocyte and biliary genes as assessed by qRT-PCR following ectopic *Sox9* expression in hepatocytes (n = 2-3). *Sox4*-expressing hepatocytes at 7 dpi (used in **Fig. 2e**) are shown as reference. In this data set, AAV8-TBG-*HA-Sox9* was injected at the dose twice that used for **Fig. 2e** (1 × 10^12^ gc/mouse) to increase the chance to find any phenotypic changes caused by *Sox9*. Data points for the empty vector (EV)-injected control are combined from 4, 7 and 14 dpi (n = 6 total).
3. Body weight changes following *Sox4* expression in hepatocytes. Data are shown as mean ± SE (n = 7 per group).
4. Representative macroscopic liver images of an AAV-*HA*-*Sox4*-injected mouse and a control empty vector-injected mouse at 7 dpi.
5. Kinetics of protein expression of the early-to-intermediate reprogramming marker CD24 and the intermediate-to-late reprogramming marker EPCAM as assessed by flow cytometry using the whole liver cells isolated at the designated time points (n = 2-3 per time point). Data points for the empty vector control are combined from 4, 7 and 10 dpi (n = 4 total).

Statistical differences were calculated by t-test. *p < 0.05, **p < 0.01, ****p<0.0001


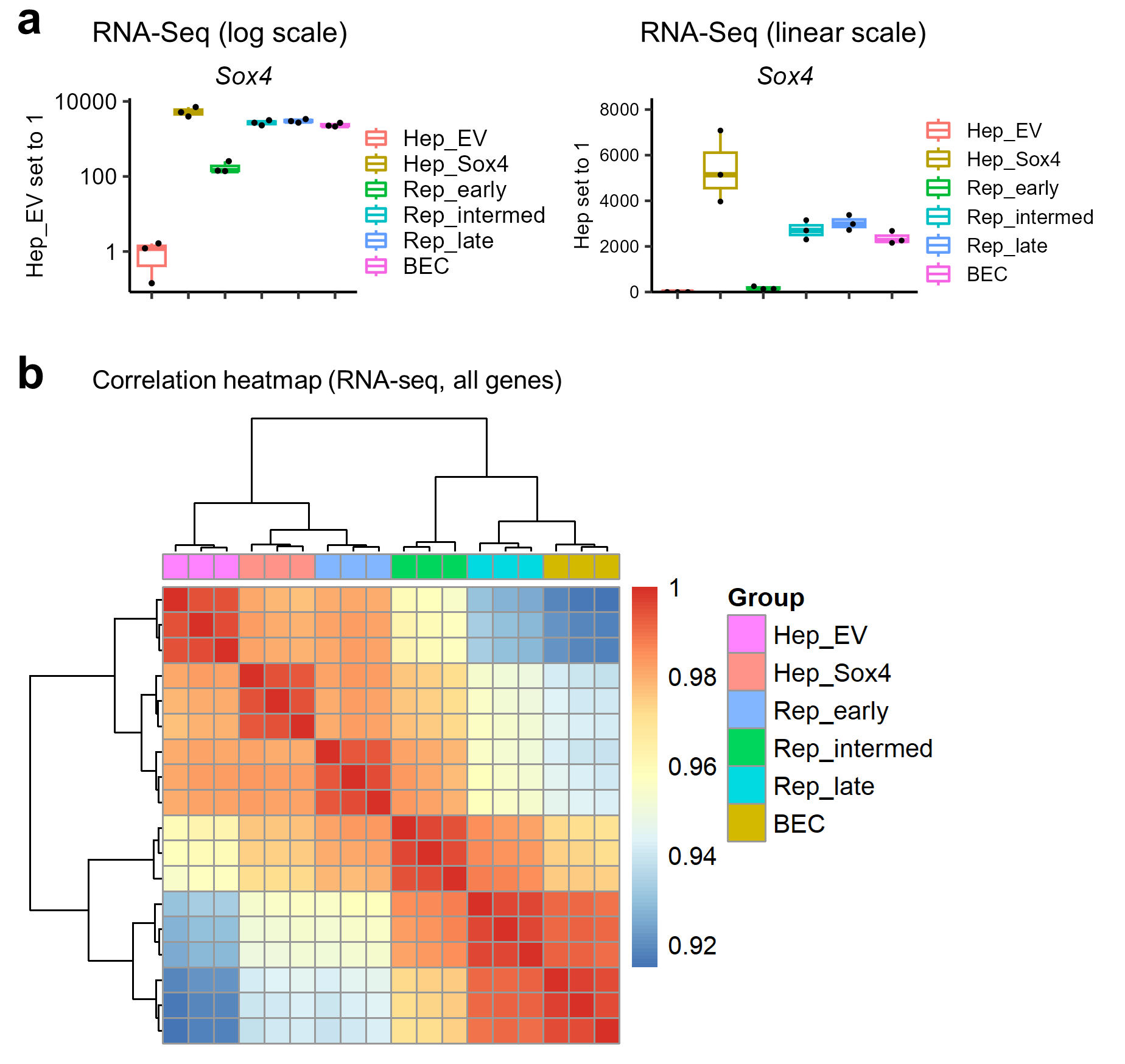


**Supplementary Fig. 8. Characterization of *Sox4*-expressing hepatocytes by RNA-Seq.**

1. Comparison of *Sox4* expression levels between ectopic *Sox4* expression system and DDC-induced reprogrammed and biliary cells by RNA-Seq.
2. Correlation heatmap of RNA-Seq of *Sox4*-expressing and EV hepatocytes in comparison to DDC-induced reprogrammed cells.


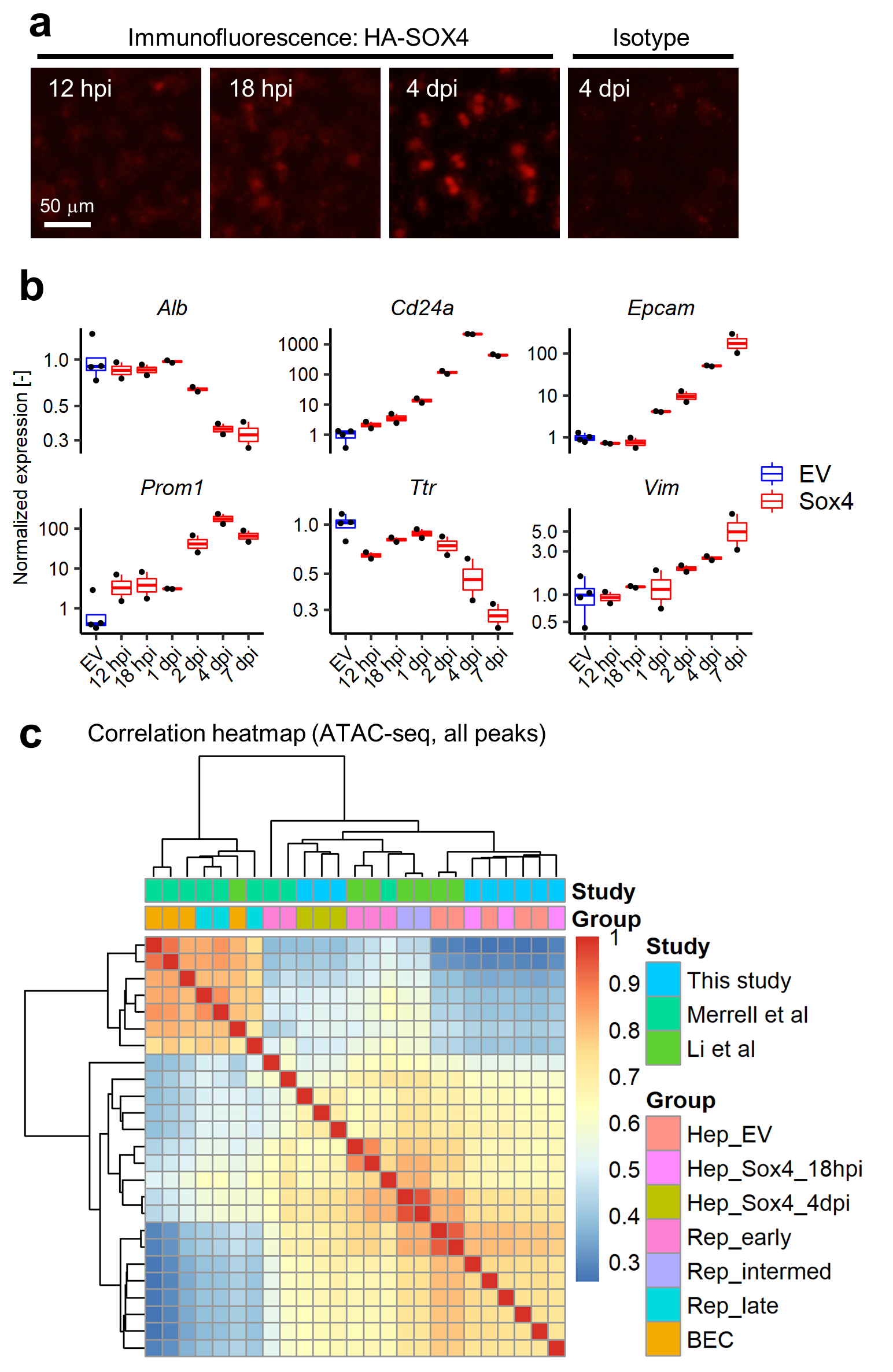


**Supplementary Fig. 9. Kinetic analysis of phenotypic changes following *Sox4* expression and the correlation heatmap of the ATAC-Seq data.**

1. Immunofluorescence staining for HA-SOX4 protein using an anti-HA-tag antibody in primary hepatocytes at the indicated timepoints following infection. Percoll-enriched hepatocytes were treated with digitonin for permeabilization, captured on a magnet using Concanavalin A-conjugated beads, and then incubated with an anti-HA antibody overnight.
2. Kinetic analysis of phenotypic changes following *Sox4* expression as assessed by qRT-PCR (n = 2 per time point for *Sox4* expressing samples). Data points for the empty vector (EV) control are combined from 4, 7 and 10 dpi (n = 4 total). Expression levels are normalized to *Actb*, with the expression level of the empty vector control set to one.
3. Correlation heatmap of ATAC-Seq of *Sox4*-expressing and EV hepatocytes in comparison with DDC-induced reprogrammed cells.


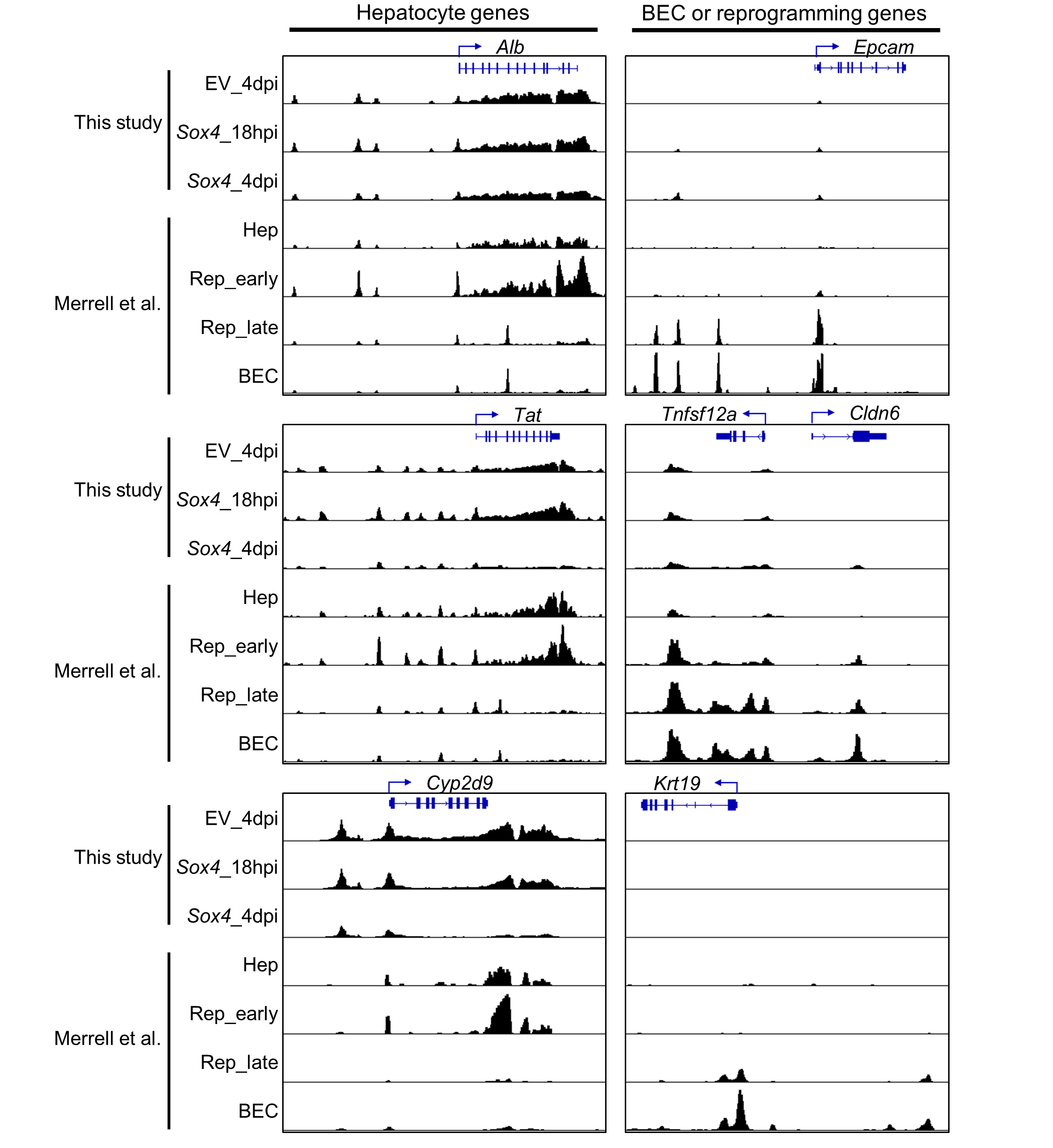


**Supplementary Fig. 10. Representative browser views of ATAC-Seq data.**

Snapshots of genome track view for ATAC-Seq at key hepatobiliary genes. Note that normal hepatocyte data from Merrell et al are not very consistent with our Hep_EV samples, presumably because the read depth of the hepatocyte samples from Merrell et al. was very low, which partly explains why these samples showed very different PC2 values from other samples in **Fig. 3e**.


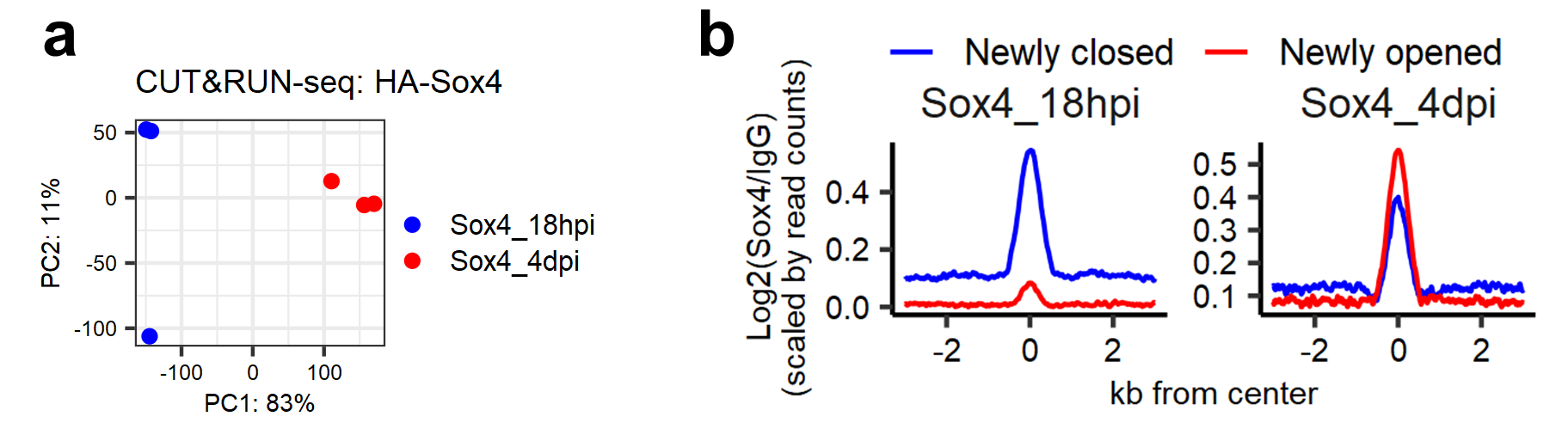


**Supplementary Fig. 11. Validation of consistency in CUT&RUN-seq data among three replicates and profiling the SOX4 binding sites in hepatocytes by CUT&RUN-Seq.**

1. PCA plots of HA-SOX4 CUT&RUN peaks at 18 hpi and 4 dpi. Peak quantification was performed using the DiffBind package.
2. Averaged aggregate plots for SOX4 CUT&RUN-Seq which are represented as the log2 ratio of SOX4 signals / IgG signals. The plots correspond to the SOX4 heatmaps shown in **Fig. 3i,** right two columns.


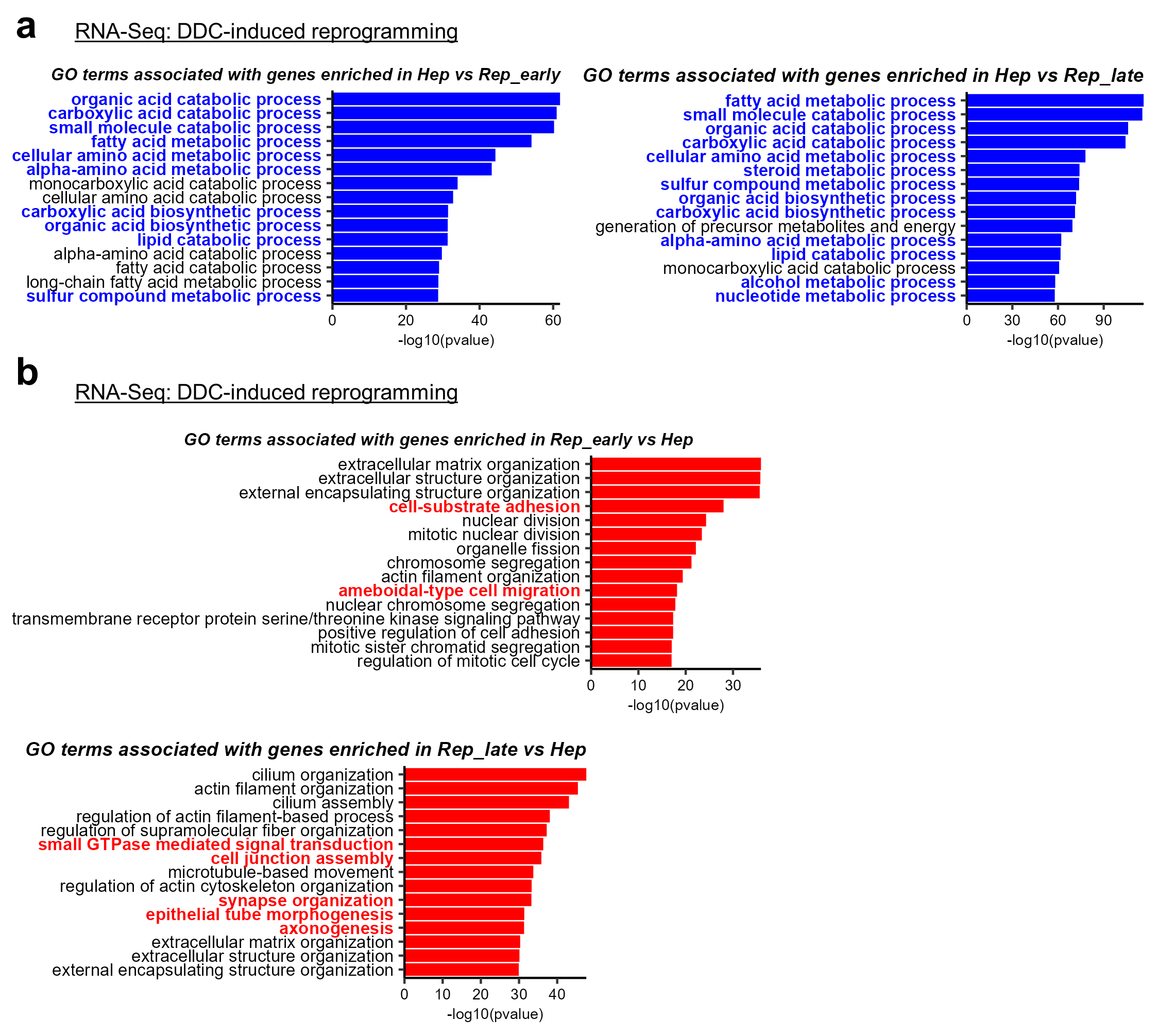


**Supplementary Fig. 12. Gene ontology (GO) analysis for upregulated or downregulated genes during DDC-induced reprogramming.**

1. GO analysis was performed using genes downregulated during DDC-induced reprogramming at different stages (RNA-Seq), namely Rep_early vs Hep (left), Rep_intermed vs Hep (**Fig. 4e**), and Rep_late vs Hep (right). Top 15 GO terms are shown. GO terms shared between the newly-closed region-associated gene set (**Fig. 4d**) and DDC-induced reprogramming context at any reprogramming stages are highlighted in bold blue texts.
2. GO analysis was performed using genes upregulated during DDC-induced reprogramming at different stages (RNA-Seq), namely Rep_early vs Hep (left), Rep_intermed vs Hep (**Fig. 4g**), and Rep_late vs Hep (right). Top 15 GO terms are shown. GO terms shared between the newly-closed region-associated gene set (**Fig. 4f**) and DDC-induced reprogramming context at any reprogramming stages are highlighted in bold red texts.


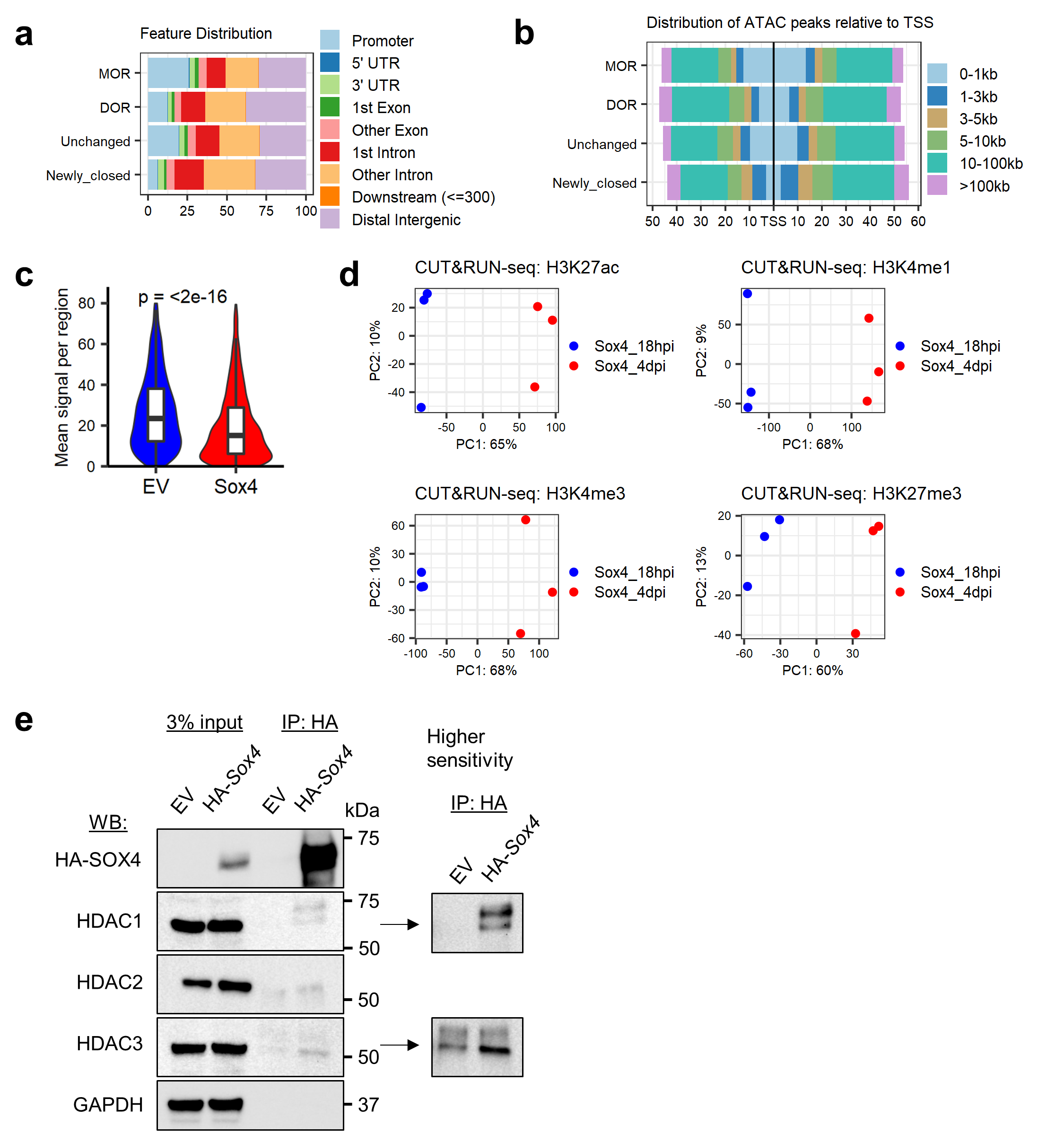


**Supplementary Fig. 13. Genomic distribution of *Sox4*-induced changes in chromatin accessibility and characterization of newly closed regions.**

1. Feature distribution of ATAC-Seq peaks at more opened regions (MORs), *de novo* opened regions (DORs), unchanged regions and newly closed regions. Promoter regions are defined as TSS ± 1 kb.
2. Distribution of the distance of ATAC-Seq peaks relative to nearest transcription start sites (TSSs).
3. The comparison of the ATAC-Seq average signals in hepatocyte enhancers (**Fig. 5g**) between empty vector and *Sox4* expressing hepatocytes. P-value is calculated by Wilcoxon rank sum test.
4. Validation of consistency in CUT&RUN-seq data among three replicates in terms of PCA mapping. Peak quantification was performed using the DiffBind package.
5. Testing protein-protein interaction of SOX4 with HDAC proteins. Hepatocytes were harvested from AAV-empty vector (EV)-injected or AAV-*HA*-*Sox4* injected mice. Immunoprecipitation (IP) using an anti-HA tag antibody was performed, and the presence of HDAC1-3 and HNF4A was assessed by western blot (WB) in the pull-down samples. GAPDH was used to validate the equality of the loading amount in terms of input (no IP) samples.


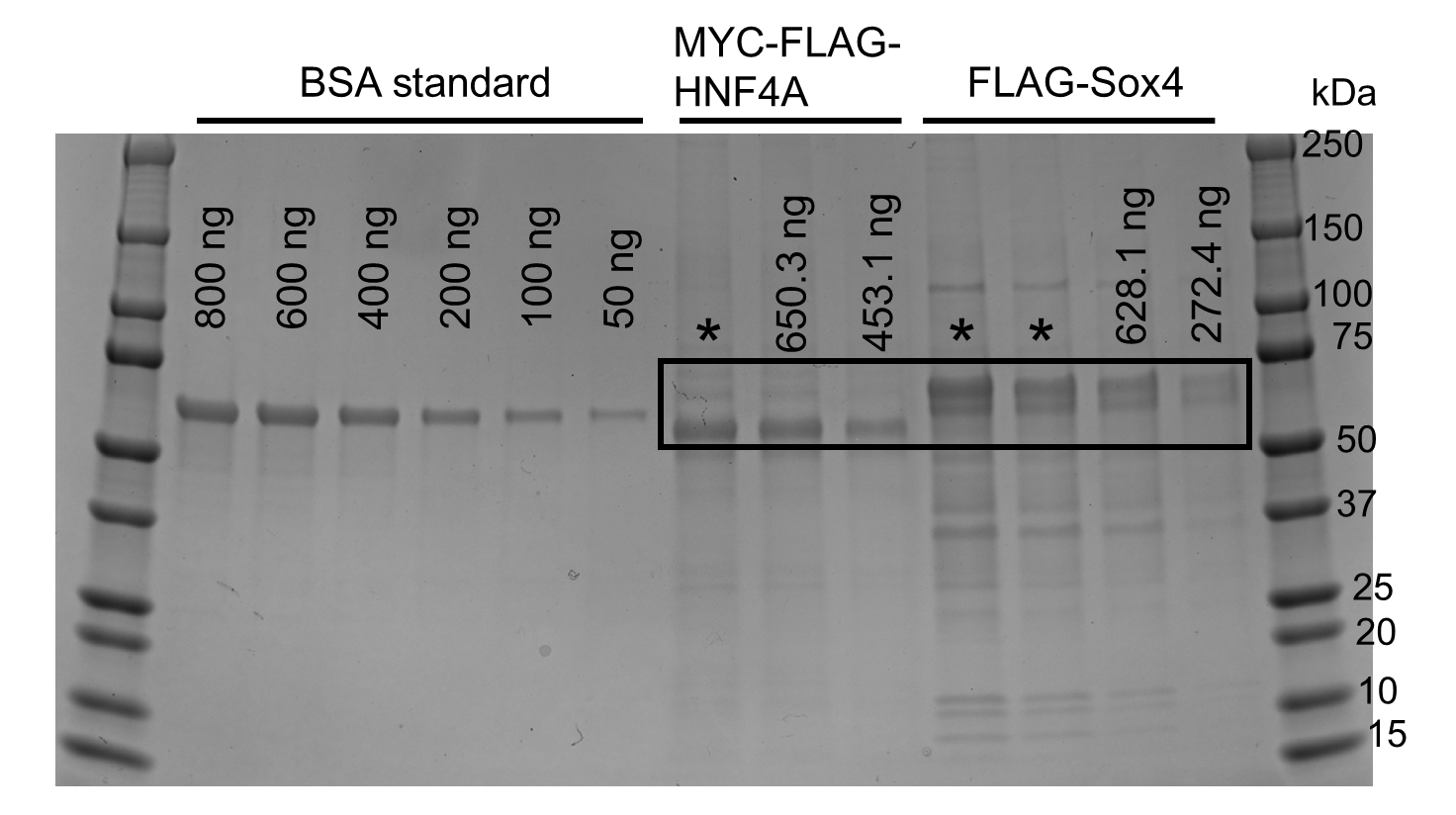


**Supplementary Fig. 14. Quality control and quantification of purified recombinant MYC-FLAG-HNF4A and FLAG-SOX4 protein by SDS-PAGE using a BSA standard.**

Rectangle region corresponds to the full-length HNF4A and SOX4 size, and used for quantification. Asterisks indicate the bands out of linear range of the BSA standards, and thus excluded from quantification.


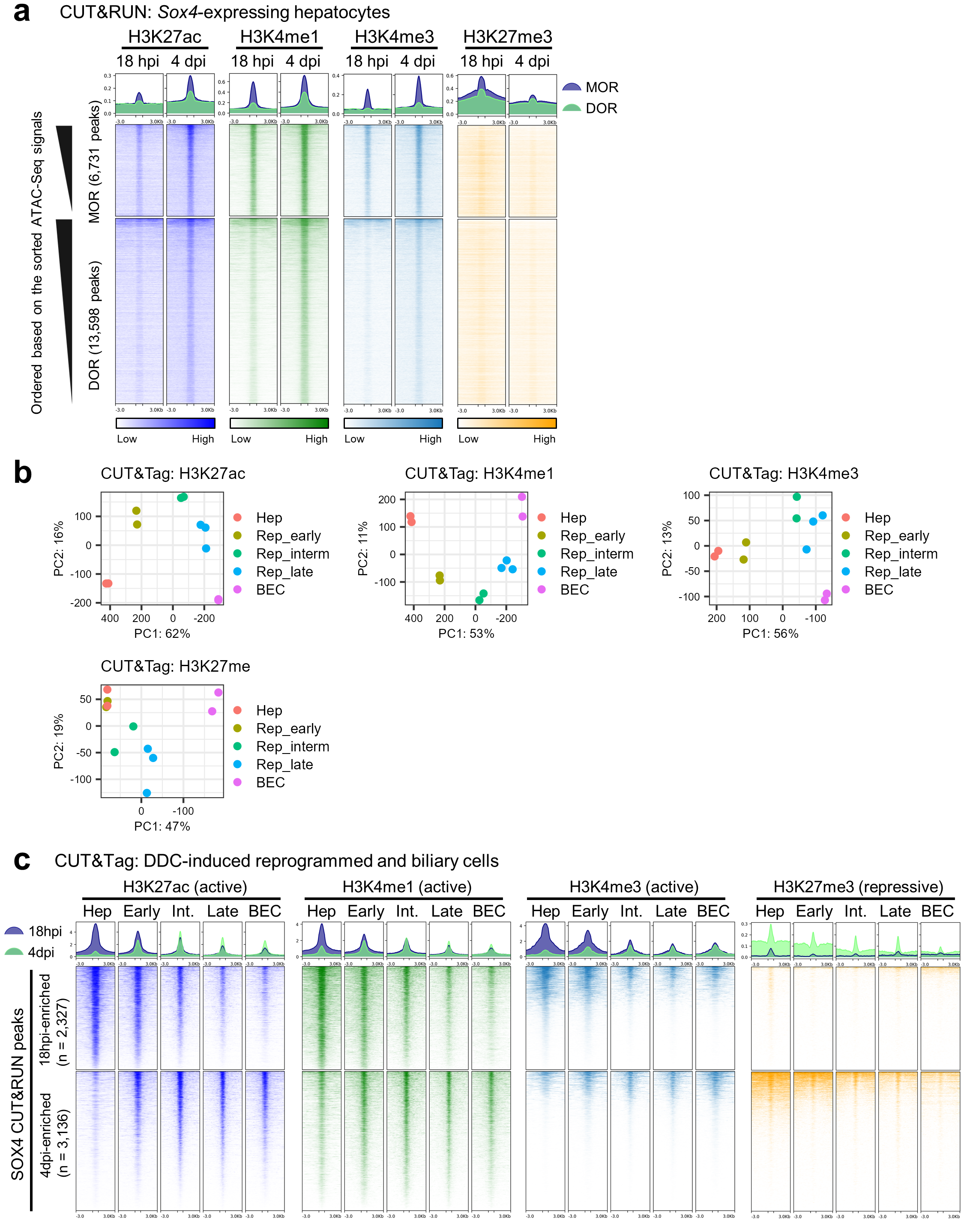


**Supplementary Fig. 15. Characterization of MORs and DORs in the ectopic *Sox4* expression and DDC-induced reprogramming contexts.**

1. CUT&RUN signals for H3K27ac, H3K4me1, H3K4me3 and H3K27me3 visualized as heatmaps for MORs and DORs. The corresponding averaged aggregate plots are shown on the top. The rows are reordered according to the signal intensities from ATAC-Seq.
2. Validation of consistency in CUT&Tag-Seq data among 2-3 replicates in terms of PCA mapping. Peak quantification was performed using the DiffBind package.
3. CUT&Tag-Seq data of histone marks shown for either 18hpi- or 4dpi-enriched Sox4 binding sites. The corresponding averaged aggregate plots are shown on the top.

**Table S1. List of known hepatocyte and biliary/reprogrammed cell genes.**

| Hepatocyte genes (source) | | Biliary/Reprogrammed cell genes (source) | |
| --- | --- | --- | --- |
| *Alb* | (Tarlow et al., 2014) | ***Ccl2*** | (Tarlow *et al.*, 2014) |
| *Cyp7a1* | (Tarlow *et al.*, 2014) | ***Zeb1*** | (Tarlow *et al.*, 2014) |
| *F9* | (Tarlow *et al.*, 2014) | ***Mst1r*** | (Tarlow *et al.*, 2014) |
| *Fah* | (Tarlow *et al.*, 2014) | ***Bmp4*** | (Tarlow *et al.*, 2014) |
| *Hgd* | (Tarlow *et al.*, 2014) | ***Vim*** | (Tarlow *et al.*, 2014) |
| *Hnf4a* | (Tarlow *et al.*, 2014) | ***Bmp1*** | (Tarlow *et al.*, 2014) |
| *Tdo2* | (Tarlow *et al.*, 2014) | ***Foxp2*** | (Tarlow *et al.*, 2014) |
| *Cps1* | (Schaub et al., 2018) | ***Sfrp4*** | (Tarlow *et al.*, 2014) |
| *Cyp26a1* | (Schaub *et al.*, 2018) | ***Etv4*** | (Tarlow *et al.*, 2014) |
| *Cyp27a1* | (Schaub *et al.*, 2018) | ***Dab1*** | (Tarlow *et al.*, 2014) |
| *Cyp2c38* | (Schaub *et al.*, 2018) | ***Kit*** | (Tarlow *et al.*, 2014) |
| *Cyp2c67* | (Schaub *et al.*, 2018) | ***Nes*** | (Tarlow *et al.*, 2014) |
| *Cyp2d10* | (Schaub *et al.*, 2018) | ***Ncam1*** | (Tarlow *et al.*, 2014) |
| *Cyp2d11* | (Schaub *et al.*, 2018) | ***Tbx1*** | (Tarlow *et al.*, 2014) |
| *Cyp2d12* | (Schaub *et al.*, 2018) | ***Mcam*** | (Tarlow *et al.*, 2014) |
| *Cyp2d13* | (Schaub *et al.*, 2018) | ***Tgfb1*** | (Tarlow *et al.*, 2014) |
| *Cyp2d40* | (Schaub *et al.*, 2018) | ***Fzd10*** | (Tarlow *et al.*, 2014) |
| *Cyp2d9* | (Schaub *et al.*, 2018) | ***Cav1*** | (Tarlow *et al.*, 2014) |
| *Cyp2j5* | (Schaub *et al.*, 2018) | ***Yap1*** | (Tarlow *et al.*, 2014) |
| *Cyp2r1* | (Schaub *et al.*, 2018) | ***Cd44*** | (Tarlow *et al.*, 2014) |
| *Cyp3a11* | (Schaub *et al.*, 2018) | ***Jag1*** | (Tarlow *et al.*, 2014) |
| *Cyp4f13* | (Schaub *et al.*, 2018) | ***Hnf1b*** | (Tarlow *et al.*, 2014) |
| *Cyp4v3* | (Schaub *et al.*, 2018) | ***Spp1*** | (Tarlow *et al.*, 2014) |
| *Cyp8b1* | (Schaub *et al.*, 2018) | ***Smo*** | (Tarlow *et al.*, 2014) |
| *Foxa3* | (Schaub *et al.*, 2018) | ***Itga3*** | (Tarlow *et al.*, 2014) |
| *Hnf1a* | (Schaub *et al.*, 2018) | ***Notch2*** | (Tarlow *et al.*, 2014) |
| *Mup1* | (Schaub *et al.*, 2018) | ***Foxj1*** | (Tarlow *et al.*, 2014) |
| *Otc* | (Schaub *et al.*, 2018) | ***Sox9*** | (Tarlow *et al.*, 2014) |
| *Tat* | (Schaub *et al.*, 2018) | ***Ccn1*** | (Tarlow *et al.*, 2014) |
| *Trf* | (Schaub *et al.*, 2018) | ***Tnfrsf12a*** | (Tarlow *et al.*, 2014) |
| *Ttr* | (Schaub *et al.*, 2018) | ***Krt19*** | (Tarlow *et al.*, 2014) |
| *Abcb11* | (Katsuda et al., 2020) | ***Grhl2*** | (Tarlow *et al.*, 2014) |
| *Ahr* | (Katsuda *et al.*, 2020) | ***Cftr*** | (Tarlow *et al.*, 2014) |
| *Aldh1a1* | (Katsuda *et al.*, 2020) | ***Epcam*** | (Tarlow *et al.*, 2014) |
| *Ass1* | (Katsuda *et al.*, 2020) | ***Krt7*** | (Tarlow *et al.*, 2014) |
| *Baat* | (Katsuda *et al.*, 2020) | ***Tacstd2*** | (Tarlow *et al.*, 2014) |
| *Cyp1a2* | (Katsuda *et al.*, 2020) | ***Ccn2*** | (Tarlow *et al.*, 2014) |
| *Cyp2e1* | (Katsuda *et al.*, 2020) | ***Krt17*** | (Schaub *et al.*, 2018) |
| *G6pc* | (Katsuda *et al.*, 2020) | ***Prom1*** | (Schaub *et al.*, 2018) |
| *Gsta1* | (Katsuda *et al.*, 2020) | ***Sstr2*** | (Schaub *et al.*, 2018) |
| *Gys2* | (Katsuda *et al.*, 2020) | ***St14*** | (Schaub *et al.*, 2018) |
| *Nr1i2* | (Katsuda *et al.*, 2020) | ***Cldn4*** | (Schaub *et al.*, 2018) |
| *Nr1i3* | (Katsuda *et al.*, 2020) | ***Muc1*** | (Schaub *et al.*, 2018) |
| *Pck1* | (Katsuda *et al.*, 2020) | ***Sox4*** | (Poncy et al., 2015) |
| *Slc10a1* | (Katsuda *et al.*, 2020) | ***Ezr*** | (Merrell et al., 2021) |
| *Serpina7* | (Katsuda *et al.*, 2020) | ***Cd24a*** | (Merrell *et al.*, 2021) |
| *Cebpa* | (Jakobsen et al., 2013) | ***Igfbp7*** | (Merrell *et al.*, 2021) |
| *Cebpb* | (Jakobsen *et al.*, 2013) | ***Itga6*** | (Yanger et al., 2013) |
| *Asgr1* | (Peters et al., 2016) | ***Cadm1*** | (Ito et al., 2007) |
| *Fabp1* | (Huang et al., 2016) |  |  |

| **Table S2. PCR primers used for cloning by NEBuilder assembly.** | | |
| --- | --- | --- |
| Construct | Block Fw/Rv | Primer sequence |
| AAV-*HA*-*Sox4*-P2A-*Cre* | Block-1 Fw | GGTGTCCAGGCGGCCACCATGTACCCATACGATGTTCCAGATTACGCTATGGTACAACAGACCAACAACGC |
|  | Block-1 Rv | CTTCAGCAGGCTGAAGTTAGTAGCTCCGCTTCCGTAGGTGAAGACCAGGTTAGAGATGC |
|  | Block-2 Fw | CTAACTTCAGCCTGCTGAAGCAGGCTGGCGACGTGGAGGAGAACCCTGGACCTCCCAAGAAGAAGAGGAAGGTGTCC |
|  | Block-2 Rv | TGTAATCCAGAGGTTGATTGTTAGTCACCATCTTCGAGCAGTCTC |
| AAV-*HA*-*Sox9*-P2A-*Cre* | Block-1 Fw | GTGTCCAGGCGGCCGCCATGTACCCATACGATGTTCCAGATTACGCTATGAATCTCCTGGACCCCTTCA |
|  | Block-1 Rv | CTTCAGCAGGCTGAAGTTAGTAGCTCCGCTTCCGGGTCTGGTGAGCTGTGTGT |
|  | Block-2 Fw  (same as Sox4 block-2) | CTAACTTCAGCCTGCTGAAGCAGGCTGGCGACGTGGAGGAGAACCCTGGACCTCCCAAGAAGAAGAGGAAGGTGTCC |
|  | Block-2 Rv  (same as Sox4 block-2) | TGTAATCCAGAGGTTGATTGTTAGTCACCATCTTCGAGCAGTCTC |
| AAV-*FLAG*-*Sox4*-P2A-*Cre* | Block-1 Fw | GGTGTCCAGGCGGCCACCATGGACTACAAAGACGATGACGACAAGATGGTACAACAGACCAACAACGC |
|  | Block-1 Rv  (same as Sox4 block-1 Fw) | CTTCAGCAGGCTGAAGTTAGTAGCTCCGCTTCCGTAGGTGAAGACCAGGTTAGAGATGC |
|  | Block-2 Fw  (same as Sox4 block-2) | CTAACTTCAGCCTGCTGAAGCAGGCTGGCGACGTGGAGGAGAACCCTGGACCTCCCAAGAAGAAGAGGAAGGTGTCC |
|  | Block-2 Rv  (same as Sox4 block-2) | TGTAATCCAGAGGTTGATTGTTAGTCACCATCTTCGAGCAGTCTC |

| Table S3. Antibodies used in this study. | | | | | |  |  |
| --- | --- | --- | --- | --- | --- | --- | --- |
| Antibody | Host | Catalog # | Dilution | Manufacturer | Application | | |
| PE/Cy7-Cd11b | Rat | 101216 | 1:100 | BioLegend | Flow / FACS | | |
| PE/Cy7-Cd31 | Rat | 102418 | 1:100 | BioLegend | Flow / FACS | | |
| PE/Cy7-Cd45 | Rat | 103114 | 1:100 | BioLegend | Flow / FACS | | |
| BV421-Cd24 | Rat | 101826 | 1:100 | BioLegend | Flow / FACS | | |
| BV421-Epcam | Rat | 118225 | 1:100 | BioLegend | Flow / FACS | | |
| PE/Dazzle594-Epcam | Rat | 118236 | 1:100 | BioLegend | Flow / FACS | | |
| Cd11b | Rat | 101202 | 1:100 | BioLegend | Dynabeads for NPC removal | | |
| Cd31 | Rat | 102402 | 1:100 | BioLegend | Dynabeads for NPC removal | | |
| Cd45 | Rat | 103102 | 1:100 | BioLegend | Dynabeads for NPC removal | | |
| Dynabeads™ anti-Rat IgG | Sheep | 11035 | 1:10 | Thermo | Dynabeads for NPC removal | | |
| MicroBeads-Epcam | Rat | 130-105-958 | 1:11 | Miltenyi | MACS for Epcam+ cell enrichment | | |
| GFP | Goat | ab6673 | 1:500 | Abcam | Immunofluorescence | | |
| Cd24 | Rat | 101801 | 1:100 | BioLegend | Immunofluorescence | | |
| Prom1 | Rat | 14-1331-80 | 1:100 | eBioscience | Immunofluorescence | | |
| Itga6 | Rat | N/A | 1:100 | V. Factor Lab | Immunofluorescence | | |
| Epcam | Rabbit | 50591-R002 | 1:500 | Sino Biological | Immunofluorescence | | |
| Krt19 | Rabbit | N/A | 1:1000 | In-house | Immunofluorescence | | |
| HA-Tag | Rabbit | 3724S | 1:1000 | CST | Immunofluorescence | | |
| Isotype control | Rabbit | 3900S | 1:1000 | CST | Immunofluorescence | | |
| AlexaFluor488-anti-goat IgG | Donkey | A-11055 | 1:300 | Invitrogen | Immunofluorescence | | |
| AlexaFluor594-anti-rabbit IgG | Donkey | A-21207 | 1:300 | Invitrogen | Immunofluorescence | | |
| AlexaFluor594-anti-rat IgG | Donkey | A-21209 | 1:300 | Invitrogen | Immunofluorescence | | |
| AlexaFluor-647-anti-rabbit IgG | Donkey | A-31573 | 1:300 | Invitrogen | Immunofluorescence | | |
| HA-Tag | Rabbit | 3724S | 1:100 | CST | CUT&RUN-Seq | | |
| Isotype control | Rabbit | 3900S | 1:100 | CST | CUT&RUN-Seq | | |
| H3K27ac | Rabbit | ab4729 | 1:100 | Abcam | CUT&RUN-Seq  CUT&Tag-Seq | | |
| H3K27me3 | Rabbit | 9733 | 1:100 | CST | CUT&RUN-Seq  CUT&Tag-Seq | | |
| H3K4me1 | Rabbit | ab8895 | 1:100 | Abcam | CUT&RUN-Seq  CUT&Tag-Seq | | |
| H3K4me3 | Rabbit | ab8580 | 1:100 | Abcam | CUT&RUN-Seq  CUT&Tag-Seq | | |
| HA-Tag | Rabbit | 3724S | 1:1000 | CST | Western blot | | |
| HDAC1 | Rabbit | 34589 | 1:1000 | CST | Western blot | | |
| HDAC2 | Rabbit | 57156 | 1:1000 | CST | Western blot | | |
| HDAC3 | Rabbit | 85057 | 1:1000 | CST | Western blot | | |
| GAPDH | Rabbit | 2118 | 1:5000 | CST | Western blot | | |

| **Table S4. Primers used for qRT-PCR.** | | |
| --- | --- | --- |
| Target | Forward | Reverse |
| *Ctgf* | GGGCCTCTTCTGCGATTTC | ATCCAGGCAAGTGCATTGGTA |
| *Cd44* | TCGATTTGAATGTAACCTGCCG | CAGTCCGGGAGATACTGTAGC |
| *Vim* | CGTCCACACGCACCTACAG | GGGGGATGAGGAATAGAGGCT |
| *Spp1* | GCTTGGCTTATGGACTGAGG | CGCTCTTCATGTGAGAGGTG |
| *Epcam* | TCTACAAGGAAGAAATCAGCAAAA | CCCTCCTCAGTTCAGCACTC |
| *Cftr* | TGCACAGTCATCCTCTGTGA | AAGGGAGTCGTACTGCCAGA |
| *Prom1* | CTGCCCAAGCTGGAAGAATA | AGCCCAGGAAAAAGAAGGTC |
| *Sox4* | CCTCGCTCTCCTCGTCCT | TCGTCTTCGAACTCGTCGT |
| *Cd24a* | CTTCTGGCACTGCTCCTACC | TACTTGGATTTGGGGAAGCA |
| *Itga6* | TCATCCTCCTGGCTGTTCTT | GTATCGGGGAATGCTGTCAT |
| *Krt7* | CATTGAGATCGCCACCTACC | GATAAGCTTGCCACCATTGC |
| *Krt19* | TTGAGAGCCTGAAGGAGGAG | AATCCACCTCCACACTGACC |
| *Cav1* | GCGACCCCAAGCATCTCAA | ATGCCGTCGAAACTGTGTGT |
| *Itga3* | CCTCTTCGGCTACTCGGTC | CCAGTCCGGTTGGTATAGTCATC |
| *Sox9* | GACTCCCCACATTCCTCCTC | CCCTCTCGCTTCAGATCAAC |
| *Hnf1b* | TCTCACCAGCATGTCTTCCA | AAAATGGGGTCCTTGTTGCT |
| *Asgr1* | TTGGATTGGCCTAACTGACC | GCCCATGTCCGTACCAGTTA |
| *Tdo2* | GGGGATCCTCAGGCTATCAT | TACCCAGTGTCTGGGAACCA |
| *Ttr* | TGGACACCAAATCGTACTGG | CAGAGTCGTTGGCTGTGAAA |
| *Fah* | CGGCGATGAAGTCATCATAA | GAGCTTCAGGCTGGTGAAAG |
| *Cebpa* | CTCCCAGAGGACCAATGAAA | AAGTCTTAGCCGGAGGAAGC |
| *Hnf4a* | GCCTCAAAGCCATCATCTTC | CCGGTCGTTGATGTAATCCT |
| *Alb* | GCTGAGACCTTCACCTTCCA | CTTGTGCTTCACCAGCTCAG |
| *G6pc* | CTGTGCAGCTGAACGTCTGT | GAAAGTTTCAGCCACAGCAA |

**References**

Huang, H., McIntosh, A.L., Martin, G.G., Landrock, D., Chung, S., Landrock, K.K., Dangott, L.J., Li, S., Kier, A.B., and Schroeder, F. (2016). FABP1: A Novel Hepatic Endocannabinoid and Cannabinoid Binding Protein. Biochemistry *55*, 5243-5255. 10.1021/acs.biochem.6b00446.

Ito, A., Nishikawa, Y., Ohnuma, K., Ohnuma, I., Koma, Y., Sato, A., Enomoto, K., Tsujimura, T., and Yokozaki, H. (2007). SgIGSF is a novel biliary-epithelial cell adhesion molecule mediating duct/ductule development. Hepatology *45*, 684-694. 10.1002/hep.21501.

Jakobsen, J.S., Waage, J., Rapin, N., Bisgaard, H.C., Larsen, F.S., and Porse, B.T. (2013). Temporal mapping of CEBPA and CEBPB binding during liver regeneration reveals dynamic occupancy and specific regulatory codes for homeostatic and cell cycle gene batteries. Genome Res *23*, 592-603. 10.1101/gr.146399.112.

Katsuda, T., Hosaka, K., Matsuzaki, J., Usuba, W., Prieto-Vila, M., Yamaguchi, T., Tsuchiya, A., Terai, S., and Ochiya, T. (2020). Transcriptomic Dissection of Hepatocyte Heterogeneity: Linking Ploidy, Zonation, and Stem/Progenitor Cell Characteristics. Cell Mol Gastroenterol Hepatol *9*, 161-183. 10.1016/j.jcmgh.2019.08.011.

Merrell, A.J., Peng, T., Li, J., Sun, K., Li, B., Katsuda, T., Grompe, M., Tan, K., and Stanger, B.Z. (2021). Dynamic Transcriptional and Epigenetic Changes Drive Cellular Plasticity in the Liver. Hepatology *74*, 444-457. 10.1002/hep.31704.

Peters, D.T., Henderson, C.A., Warren, C.R., Friesen, M., Xia, F., Becker, C.E., Musunuru, K., and Cowan, C.A. (2016). Asialoglycoprotein receptor 1 is a specific cell-surface marker for isolating hepatocytes derived from human pluripotent stem cells. Development *143*, 1475-1481. 10.1242/dev.132209.

Poncy, A., Antoniou, A., Cordi, S., Pierreux, C.E., Jacquemin, P., and Lemaigre, F.P. (2015). Transcription factors SOX4 and SOX9 cooperatively control development of bile ducts. Dev Biol *404*, 136-148. 10.1016/j.ydbio.2015.05.012.

Schaub, J.R., Huppert, K.A., Kurial, S.N.T., Hsu, B.Y., Cast, A.E., Donnelly, B., Karns, R.A., Chen, F., Rezvani, M., Luu, H.Y., et al. (2018). De novo formation of the biliary system by TGFbeta-mediated hepatocyte transdifferentiation. Nature *557*, 247-251. 10.1038/s41586-018-0075-5.

Tarlow, B.D., Pelz, C., Naugler, W.E., Wakefield, L., Wilson, E.M., Finegold, M.J., and Grompe, M. (2014). Bipotential adult liver progenitors are derived from chronically injured mature hepatocytes. Cell Stem Cell *15*, 605-618. 10.1016/j.stem.2014.09.008.

Yanger, K., Zong, Y., Maggs, L.R., Shapira, S.N., Maddipati, R., Aiello, N.M., Thung, S.N., Wells, R.G., Greenbaum, L.E., and Stanger, B.Z. (2013). Robust cellular reprogramming occurs spontaneously during liver regeneration. Genes Dev *27*, 719-724. 10.1101/gad.207803.112.

**Additional Source Material: Uncropped blots for Supplementary Fig. 13e**

|  |  | | |  | | |  | | |  |  | |  | |  | | |  | |  | |  |  |  |  |  |
| --- | --- | --- | --- | --- | --- | --- | --- | --- | --- | --- | --- | --- | --- | --- | --- | --- | --- | --- | --- | --- | --- | --- | --- | --- | --- | --- |
|  | |  | | |  | | |  | | | |  | |  | |  |  | |  | |  | | |  |  |  |
|  | |  |  | | |  | | |  |  |  |  |  |  |  |  |  |  |  |  |  |  |  |  |  |  |
|  | |  | | |  | | |  | | | |  | |  | |  |  | |  | |  | | |  |  |  |
|  | | 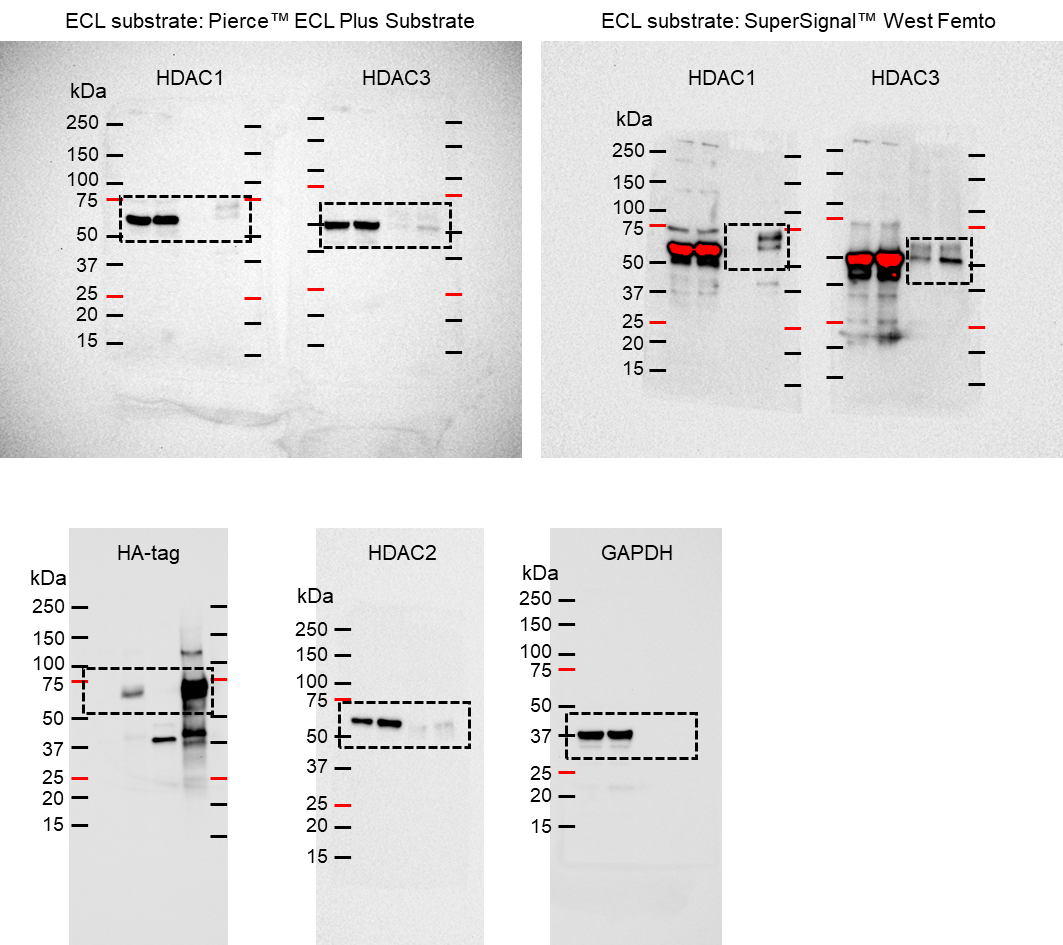 | | | | | | | | | | | | | | | | | | | | | |  |  |  |
|  | |  |  |  |  |  |  |  |  |  |  |  |  |  |  |  |  |  |  |  |  |  |  |  |  |  |
|  | |  |  |  |  |  |  |  |  |  |  |  |  |  |  |  |  |  |  |  |  |  |  |  |  |  |
|  | |  |  |  |  |  |  |  |  |  |  |  |  |  |  |  |  |  |  |  |  |  |  |  |  |  |
|  | |  |  |  |  |  |  |  |  |  |  |  |  |  |  |  |  |  |  |  |  |  |  |  |  |  |
|  | |  |  |  |  |  |  |  |  |  |  |  |  |  |  |  |  |  |  |  |  |  |  |  |  |  |
|  | |  |  |  |  |  |  |  |  |  |  |  |  |  |  |  |  |  |  |  |  |  |  |  |  |  |
|  | |  |  |  |  |  |  |  |  |  |  |  |  |  |  |  |  |  |  |  |  |  |  |  |  |  |
|  | |  |  |  |  |  |  |  |  |  |  |  |  |  |  |  |  |  |  |  |  |  |  |  |  |  |
|  | |  |  |  |  |  |  |  |  |  |  |  |  |  |  |  |  |  |  |  |  |  |  |  |  |  |
|  | |  |  |  |  |  |  |  |  |  |  |  |  |  |  |  |  |  |  |  |  |  |  |  |  |  |
|  | |  |  |  |  |  |  |  |  |  |  |  |  |  |  |  |  |  |  |  |  |  |  |  |  |  |
|  | |  |  |  |  |  |  |  |  |  |  |  |  |  |  |  |  |  |  |  |  |  |  |  |  |  |
|  | |  |  |  |  |  |  |  |  |  |  |  |  |  |  |  |  |  |  |  |  |  |  |  |  |  |
|  | |  |  |  |  |  |  |  |  |  |  |  |  |  |  |  |  |  |  |  |  |  |  |  |  |  |
|  | |  |  |  |  |  |  |  |  |  |  |  |  |  |  |  |  |  |  |  |  |  |  |  |  |  |
|  | |  |  |  |  |  |  |  |  |  |  |  |  |  |  |  |  |  |  |  |  |  |  |  |  |  |
|  | |  |  |  |  |  |  |  |  |  |  |  |  |  |  |  |  |  |  |  |  |  |  |  |  |  |
|  | |  |  |  |  |  |  |  |  |  |  |  |  |  |  |  |  |  |  |  |  |  |  |  |  |  |
|  | |  |  |  |  |  |  |  |  |  |  |  |  |  |  |  |  |  |  |  |  |  |  |  |  |  |
|  | |  |  |  |  |  |  |  |  |  |  |  |  |  |  |  |  |  |  |  |  |  |  |  |  |  |
|  | |  |  |  |  |  |  |  |  |  |  |  |  |  |  |  |  |  |  |  |  |  |  |  |  |  |
|  | |  |  |  |  |  |  |  |  |  |  |  |  |  |  |  |  |  |  |  |  |  |  |  |  |  |
|  | |  |  |  |  |  |  |  |  |  |  |  |  |  |  |  |  |  |  |  |  |  |  |  |  |  |
|  | |  |  |  |  |  |  |  |  |  |  |  |  |  |  |  |  |  |  |  |  |  |  |  |  |  |
|  | |  |  |  |  |  |  |  |  |  |  |  |  |  |  |  |  |  |  |  |  |  |  |  |  |  |
