# Supplementary material for: Cellular reprogramming in vivo initiated by SOX4 pioneer factor activity
Source: Nat Commun. 2024 Feb 26;15:1761. doi: 10.1038/s41467-024-45939-z (PMC10897393; doi:10.1038/s41467-024-45939-z)
Supplement: Supplementary file 3 — Description of Additional Supplementary Files [file 41467_2024_45939_MOESM3_ESM.pdf]

Description of Additional Supplementary Files

File Name: Supplementary Data 1
Description: Differentially expressed genes between hepatocytes and early reprogrammed cells.

File Name: Supplementary Data 2
Description: GO terms associated with genes near newly opened and newly closed regions.

File Name: Supplementary Data 3
Description: GO terms associated with pairwise reprogramming comparisons.
