# Supplementary material for: Cellular reprogramming in vivo initiated by SOX4 pioneer factor activity
Source: Nat Commun. 2024 Feb 26;15:1761. doi: 10.1038/s41467-024-45939-z (PMC10897393; doi:10.1038/s41467-024-45939-z)
Supplement: Supplementary file 7 — Reporting Summary [file 41467_2024_45939_MOESM7_ESM.pdf]

nature portfolio

Corresponding author(s): Ben Stanger
Last updated by author(s): 1/21/2024

Reporting Summary

Nature Portfolio wishes to improve the reproducibility of the work that we publish. This form provides structure for consistency and transparency in reporting. For further information on Nature Portfolio policies, see our Editorial Policies and the Editorial Policy Checklist.

Statistics

For all statistical analyses, confirm that the following items are present in the figure legend, table legend, main text, or Methods section.

n/a	Confirmed	
☐	☒	The exact sample size (*n*) for each experimental group/condition, given as a discrete number and unit of measurement
☐	☒	A statement on whether measurements were taken from distinct samples or whether the same sample was measured repeatedly
☐	☒	The statistical test(s) used AND whether they are one- or two-sided<br>*Only common tests should be described solely by name; describe more complex techniques in the Methods section.*
☒	☐	A description of all covariates tested
☐	☒	A description of any assumptions or corrections, such as tests of normality and adjustment for multiple comparisons
☐	☒	A full description of the statistical parameters including central tendency (e.g. means) or other basic estimates (e.g. regression coefficient) AND variation (e.g. standard deviation) or associated estimates of uncertainty (e.g. confidence intervals)
☐	☒	For null hypothesis testing, the test statistic (e.g. *F*, *t*, *r*) with confidence intervals, effect sizes, degrees of freedom and *P* value noted<br>*Give P values as exact values whenever suitable.*
☒	☐	For Bayesian analysis, information on the choice of priors and Markov chain Monte Carlo settings
☒	☐	For hierarchical and complex designs, identification of the appropriate level for tests and full reporting of outcomes
☒	☐	Estimates of effect sizes (e.g. Cohen's *d*, Pearson's *r*), indicating how they were calculated

Our web collection on statistics for biologists contains articles on many of the points above.

Software and code

Policy information about availability of computer code

Data collection	BioRadCFXManager (qPCR)<br>Infinity Analyze (microscopic imaging)<br>Leica Application Suite (stereoscopic imaging)<br>NextSeq System Suite (NGS)<br>DivaSoftware (FACS and Flow cytometry)
Data analysis	FlowJo v10.8.1 Tree Star https://www.flowjo.com<br>ImageJ v1.53t National Institutes of Health https://imagej.nih.gov/ij/<br>Integrative Genomic Viewer (IGV) v2.14.1 Broad Institute https://software.broadinstitute.org/software/igv/<br>R v4.2.1 The R Project for Statistical Computing https://cran.r-project.org/<br>Python v3.9.12 Python Software Foundation https://www.python.org<br>Star v2.7.10a (Dobin et al., 2013) https://github.com/alexdobin/STAR<br>featureCounts v2.0.1 (Liao et al., 2014) https://subread.sourceforge.net/<br>Salmon v0.13.1 (Patro et al., 2017) https://combine-lab.github.io/salmon/<br>DESeq2 v1.36.0 (Love et al., 2014) https://lashlock.github.io/compbio/R_presentation.html<br>Bowtie2 v2.2.5 (Langmead and Salzberg, 2012) https://bowtie-bio.sourceforge.net/bowtie2/index.shtml<br>Samtools v1.6 (Li, 2011; Li et al., 2009) https://samtools.sourceforge.net/<br>Picard v2.27.4 http://broadinstitute.github.io/picard/<br>Bedtools v2.30.0 https://bedtools.readthedocs.io/en/latest/

Macs2 v2.2.7.1 (Feng et al., 2012) https://hbctraining.github.io/Intro-to-ChIPseq/lessons/05_peak_calling_macs.html
Deeptools v3.5.1 (Ramírez et al., 2016) https://deeptools.readthedocs.io/en/develop/
Homer v4.11 http://homer.ucsd.edu/homer/motif/
Tobias v0.13.3 (Bentsen et al., 2020) https://github.com/loosolab/TOBIAS
DiffBind v3.6.4 (Ross-Innes et al., 2012) https://hbctraining.github.io/Intro-to-ChIPseq/lessons/08_diffbind_differential_peaks.html
ChIPseeker v1.32.1 (Yu et al., 2015) https://rdrr.io/bioc/ChIPseeker/f/vignettes/ChIPseeker.Rmd
Fgsea v1.22.0 (Korotkevich et al.) https://github.com/ctlab/fgsea
ggupset v0.3.0 https://github.com/const-ae/ggupset
DANPOS v.3 (Chen et al., 2013) https://github.com/sklasfeld/DANPOS3

For manuscripts utilizing custom algorithms or software that are central to the research but not yet described in published literature, software must be made available to editors and reviewers. We strongly encourage code deposition in a community repository (e.g. GitHub). See the Nature Portfolio guidelines for submitting code & software for further information.

Data

Policy information about availability of data

All manuscripts must include a data availability statement. This statement should provide the following information, where applicable:

- Accession codes, unique identifiers, or web links for publicly available datasets
- A description of any restrictions on data availability
- For clinical datasets or third party data, please ensure that the statement adheres to our policy

Data and code availability
Data that generated during the study have been deposited in Gene Expression Ominubus (GEO) with the accession number GSE221225 (SuperSeries) with SubSeries accession numbers GSE218945 and GSE218947 (RNA-Seq); GSE219052 (ATAC-Seq); GSE221223 and GSE221224 (CUT&RUN-Seq). Reviewers can access the Superseries with the following token:
wvmjowcsbpodvwf
Detailed scripts and parameters used for each step of the analysis provided by reasonable request to the authors.

Human research participants

Policy information about studies involving human research participants and Sex and Gender in Research.

Reporting on sex and gender	N/A
Population characteristics	N/A
Recruitment	N/A
Ethics oversight	N/A

Note that full information on the approval of the study protocol must also be provided in the manuscript.

Field-specific reporting

Please select the one below that is the best fit for your research. If you are not sure, read the appropriate sections before making your selection.

☒ Life sciences ☐ Behavioural & social sciences ☐ Ecological, evolutionary & environmental sciences

For a reference copy of the document with all sections, see nature.com/documents/nr-reporting-summary-flat.pdf

Life sciences study design

All studies must disclose on these points even when the disclosure is negative.

Sample size	No sample size calculation was done.
Data exclusions	No data exclusion was done.
Replication	At least two biological replicates were used for each experiment.
Randomization	Animals were randomly allocated to each group.
Blinding	Blinding was not performed.

Reporting for specific materials, systems and methods

We require information from authors about some types of materials, experimental systems and methods used in many studies. Here, indicate whether each material, system or method listed is relevant to your study. If you are not sure if a list item applies to your research, read the appropriate section before selecting a response.

Materials & experimental systems

n/a	Involved in the study
☐	☒ Antibodies
☐	☒ Eukaryotic cell lines
☒	☐ Palaeontology and archaeology
☐	☒ Animals and other organisms
☒	☐ Clinical data
☒	☐ Dual use research of concern

Methods

n/a	Involved in the study
☐	☒ ChIP-seq
☐	☒ Flow cytometry
☒	☐ MRI-based neuroimaging

Antibodies

Antibodies used	Rat anti Cd11b PE/Cy7-conjugated Biolegend Cat# 101216<br>Rat anti Cd31 PE/Cy7-conjugated Biolegend Cat# 102418<br>Rat anti Cd45 PE/Cy7-conjugated Biolegend Cat# 103114<br>Rat anti Epcam BV421-conjugated Biolegend Cat# 118225<br>Rat anti Cd24 BV421-conjugated Biolegend Cat# 101826<br>Goat polyclonal anti GFP antibody Abcam Cat# ab6673<br>Chicken polyclonal anti GFP antibody Abcam Cat# ab13970<br>Goat polyclonal anti Osteopontin/OPN antibody R&D Cat# AF808<br>Rabbit monocolonal anti Krt19 antibody In-house N/A<br>Rat anti-mouse CD24 [M1/69] Biolegend Cat# 101801<br>Rat Cd133 (Prominin-1) Monoclonal Antibody (13A4) eBioscience Cat# 14-1331-80<br>Rat anti Itga6 V. Factor Lab N/A<br>Recombinant Anti-EpCAM Antibody, Rabbit Monoclonal Sino Biological Cat# 50591-R002<br>HA-Tag (C29F4) Rabbit mAb Cell Signaling Technology Cat# 3724S<br>Rabbit (DA1E) mAb IgG XP® Isotype Control Cell Signaling Technology Cat# 3900S<br>Anti-Histone H3 (acetyl K27) antibody - ChIP Grade Abcam Cat# ab4729<br>Tri-Methyl-Histone H3 (Lys27) (C36B11) Rabbit mAb Cell Signaling Technology Cat# 9733<br>Anti-Histone H3 (mono methyl K4) antibody - ChIP Grade Abcam Cat# ab8895<br>Anti-Histone H3 (tri methyl K4) antibody - ChIP Grade Abcam Cat# ab8580
Validation	All the commercial antibodies were validated by manufacturers.<br>The Krt19 and Itga6 antibodies weres validated in our earlier study (Yanger et al. Cell Stem Cell. 2014 Sep 4;15(3):340-349)

Eukaryotic cell lines

Policy information about cell lines and Sex and Gender in Research

Cell line source(s)	LentiX-HEK293T
Authentication	Authenticated by Clontech Laboratories, Inc
Mycoplasma contamination	Confirmed to be mycoplasma-free by the manufacturer.
Commonly misidentified lines (See ICLAC register)	We confirm that we have not used commonly misidentified lines

Animals and other research organisms

Policy information about studies involving animals; ARRIVE guidelines recommended for reporting animal research, and Sex and Gender in Research

Laboratory animals	Mus musculus, B6J.129(B6N)-Gt(ROSA)26Sortm1(CAG-cas9*,-EGFP)Fezh/J, 4-16 weeks
Wild animals	N/A
Reporting on sex	Only male mice were used.

Field-collected samples	N/A
Ethics oversight	All mouse experiment procedures used in this study were performed following the NIH guidelines. All mouse procedure protocols used in this study were in accordance with, and with the approval of, the Institutional Animal Care and Use Committee of the University of Pennsylvania. Mice were maintained in a standard 12-hour light/12-hour dark cycle with ambient temperatures 18-23C and approximately 50% humidity.

Note that full information on the approval of the study protocol must also be provided in the manuscript.

ChIP-seq

Data deposition

- [x] Confirm that both raw and final processed data have been deposited in a public database such as GEO.
- [x] Confirm that you have deposited or provided access to graph files (e.g. BED files) for the called peaks.

Data access links
May remain private before publication.

Data that generated during the study have been deposited in Gene Expression Ominubus (GEO) with accession numbers GSE221223 and GSE221224 (CUT&RUN-Seq) using the following link
https://www.ncbi.nlm.nih.gov/geo/query/acc.cgi?acc=GSE221225
Reviewers can access the Superseries with the following token: wvmjowcsbpodvwf

GSE237908 (CUT&Tag-Seq) using the following link
https://www.ncbi.nlm.nih.gov/geo/query/acc.cgi?&acc=GSE237908
Reviewers can access the Superseries with the following token: slgxgqyuxpstpyv

Files in database submission

GSM6856391 Sox4_18hpi, rep1 NextSeq 500, CUT&RUN
GSM6856392 Sox4_18hpi, rep2 NextSeq 500, CUT&RUN
GSM6856393 Sox4_18hpi, rep3 NextSeq 500, CUT&RUN
GSM6856394 Sox4_4dpi, rep1 NextSeq 500, CUT&RUN
GSM6856395 Sox4_4dpi, rep2 NextSeq 500, CUT&RUN
GSM6856396 Sox4_4dpi, rep3 NextSeq 500, CUT&RUN
GSM6856397 Isotype_18hpi, rep1 NextSeq 500, CUT&RUN
GSM6856398 Isotype_18hpi, rep2 NextSeq 500, CUT&RUN
GSM6856399 Isotype_18hpi, rep3 NextSeq 500, CUT&RUN
GSM6856400 Isotype_4dpi, rep1 NextSeq 500, CUT&RUN
GSM6856401 Isotype_4dpi, rep2 NextSeq 500, CUT&RUN
GSM6856402 Isotype_4dpi, rep3 NextSeq 500, CUT&RUN
GSM6856403 H3K27ac_18hpi, rep1 NextSeq 500, CUT&RUN
GSM6856404 H3K27ac_18hpi, rep2 NextSeq 500, CUT&RUN
GSM6856405 H3K27ac_4dpi, rep1 NextSeq 500, CUT&RUN
GSM6856406 H3K27ac_4dpi, rep2 NextSeq 500, CUT&RUN
GSM6856407 H3K4m27me3_18hpi, rep1 NextSeq 500, CUT&RUN
GSM6856408 H3K4m27me3_18hpi, rep2 NextSeq 500, CUT&RUN
GSM6856409 H3K4m27me3_4dpi, rep1 NextSeq 500, CUT&RUN
GSM6856410 H3K4m27me3_4dpi, rep2 NextSeq 500, CUT&RUN
GSM6856411 H3K4me1_18hpi, rep1 NextSeq 500, CUT&RUN
GSM6856412 H3K4me1_4dpi, rep1 NextSeq 500, CUT&RUN
GSM6856413 H3K4me3_18hpi, rep1 NextSeq 500, CUT&RUN
GSM6856414 H3K4me3_4dpi, rep1 NextSeq 500, CUT&RUN
GSM6856415 Sox4_18hpi, rep1 NextSeq 2000, CUT&RUN
GSM6856416 Sox4_18hpi, rep2 NextSeq 2000, CUT&RUN
GSM6856417 Sox4_18hpi, rep3 NextSeq 2000, CUT&RUN
GSM6856418 Sox4_4dpi, rep1 NextSeq 2000, CUT&RUN
GSM6856419 Sox4_4dpi, rep2 NextSeq 2000, CUT&RUN
GSM6856420 Sox4_4dpi, rep3 NextSeq 2000, CUT&RUN
GSM6856421 Isotype_18hpi, rep1 NextSeq 2000, CUT&RUN
GSM6856422 Isotype_18hpi, rep2 NextSeq 2000, CUT&RUN
GSM6856423 Isotype_18hpi, rep3 NextSeq 2000, CUT&RUN
GSM6856424 Isotype_4dpi, rep1 NextSeq 2000, CUT&RUN
GSM6856425 Isotype_4dpi, rep2 NextSeq 2000, CUT&RUN
GSM6856426 Isotype_4dpi, rep3 NextSeq 2000, CUT&RUN
GSM6856427 H3K27ac_18hpi, rep3 NextSeq 2000, CUT&RUN
GSM6856428 H3K27ac_4dpi, rep3 NextSeq 2000, CUT&RUN
GSM6856429 H3K4m27me3_18hpi, rep3 NextSeq 2000, CUT&RUN
GSM6856430 H3K4m27me3_4dpi, rep3 NextSeq 2000, CUT&RUN
GSM6856431 H3K4me1_18hpi, rep2 NextSeq 2000, CUT&RUN
GSM6856432 H3K4me1_18hpi, rep3 NextSeq 2000, CUT&RUN
GSM6856433 H3K4me1_4dpi, rep2 NextSeq 2000, CUT&RUN

GSM6856434 H3K4me1_4dpi, rep3 NextSeq 2000, CUT&RUN
GSM6856435 H3K4me3_18hpi, rep2 NextSeq 2000, CUT&RUN
GSM6856436 H3K4me3_18hpi, rep3 NextSeq 2000, CUT&RUN
GSM6856437 H3K4me3_4dpi, rep2 NextSeq 2000, CUT&RUN
GSM6856438 H3K4me3_4dpi, rep3 NextSeq 2000, CUT&RUN
GSM7656257 BEC_rep1_H3K27ac NextSeq2000, CUT&Tag
GSM7656268 BEC_rep1_H3K27me3 NextSeq2000, CUT&Tag
GSM7656235 BEC_rep1_H3K4me1 NextSeq2000, CUT&Tag
GSM7656246 BEC_rep1_H3K4me3 NextSeq2000, CUT&Tag
GSM7656258 BEC_rep2_H3K27ac NextSeq2000, CUT&Tag
GSM7656269 BEC_rep2_H3K27me3 NextSeq2000, CUT&Tag
GSM7656236 BEC_rep2_H3K4me1 NextSeq2000, CUT&Tag
GSM7656247 BEC_rep2_H3K4me3 NextSeq2000, CUT&Tag
GSM7656248 Hep_rep1_H3K27ac NextSeq2000, CUT&Tag
GSM7656259 Hep_rep1_H3K27me3 NextSeq2000, CUT&Tag
GSM7656226 Hep_rep1_H3K4me1 NextSeq2000, CUT&Tag
GSM7656237 Hep_rep1_H3K4me3 NextSeq2000, CUT&Tag
GSM7656249 Hep_rep2_H3K27ac NextSeq2000, CUT&Tag
GSM7656260 Hep_rep2_H3K27me3 NextSeq2000, CUT&Tag
GSM7656227 Hep_rep2_H3K4me1 NextSeq2000, CUT&Tag
GSM7656238 Hep_rep2_H3K4me3 NextSeq2000, CUT&Tag
GSM7656250 Rep_early_rep1_H3K27ac NextSeq2000, CUT&Tag
GSM7656261 Rep_early_rep1_H3K27me3 NextSeq2000, CUT&Tag
GSM7656228 Rep_early_rep1_H3K4me1 NextSeq2000, CUT&Tag
GSM7656239 Rep_early_rep1_H3K4me3 NextSeq2000, CUT&Tag
GSM7656251 Rep_early_rep2_H3K27ac NextSeq2000, CUT&Tag
GSM7656262 Rep_early_rep2_H3K27me3 NextSeq2000, CUT&Tag
GSM7656229 Rep_early_rep2_H3K4me1 NextSeq2000, CUT&Tag
GSM7656240 Rep_early_rep2_H3K4me3 NextSeq2000, CUT&Tag
GSM7656252 Rep_intermed_rep1_H3K27ac NextSeq2000, CUT&Tag
GSM7656263 Rep_intermed_rep1_H3K27me3 NextSeq2000, CUT&Tag
GSM7656230 Rep_intermed_rep1_H3K4me1 NextSeq2000, CUT&Tag
GSM7656241 Rep_intermed_rep1_H3K4me3 NextSeq2000, CUT&Tag
GSM7656253 Rep_intermed_rep2_H3K27ac NextSeq2000, CUT&Tag
GSM7656264 Rep_intermed_rep2_H3K27me3 NextSeq2000, CUT&Tag
GSM7656231 Rep_intermed_rep2_H3K4me1 NextSeq2000, CUT&Tag
GSM7656242 Rep_intermed_rep2_H3K4me3 NextSeq2000, CUT&Tag
GSM7656254 Rep_late_rep1_H3K27ac NextSeq2000, CUT&Tag
GSM7656265 Rep_late_rep1_H3K27me3 NextSeq2000, CUT&Tag
GSM7656232 Rep_late_rep1_H3K4me1 NextSeq2000, CUT&Tag
GSM7656243 Rep_late_rep1_H3K4me3 NextSeq2000, CUT&Tag
GSM7656255 Rep_late_rep2_H3K27ac NextSeq2000, CUT&Tag
GSM7656266 Rep_late_rep2_H3K27me3 NextSeq2000, CUT&Tag
GSM7656233 Rep_late_rep2_H3K4me1 NextSeq2000, CUT&Tag
GSM7656244 Rep_late_rep2_H3K4me3 NextSeq2000, CUT&Tag
GSM7656256 Rep_late_rep3_H3K27ac NextSeq2000, CUT&Tag
GSM7656267 Rep_late_rep3_H3K27me3 NextSeq2000, CUT&Tag
GSM7656234 Rep_late_rep3_H3K4me1 NextSeq2000, CUT&Tag
GSM7656245 Rep_late_rep3_H3K4me3 3437933, CUT&Tag

Genome browser session
(e.g. UCSC)

N/A

Methodology

Replicates

Biological replicates obtained from 3 mice.

Sequencing depth

GSM6856391 Sox4_18hpi, rep1 NextSeq 500, CUT&RUN: 3,380,894
GSM6856392 Sox4_18hpi, rep2 NextSeq 500, CUT&RUN: 1,713,647
GSM6856393 Sox4_18hpi, rep3 NextSeq 500, CUT&RUN: 4,213,579
GSM6856394 Sox4_4dpi, rep1 NextSeq 500, CUT&RUN: 3,369,238
GSM6856395 Sox4_4dpi, rep2 NextSeq 500, CUT&RUN: 5,171,551
GSM6856396 Sox4_4dpi, rep3 NextSeq 500, CUT&RUN: 5,665,642
GSM6856397 Isotype_18hpi, rep1 NextSeq 500, CUT&RUN: 309,624
GSM6856398 Isotype_18hpi, rep2 NextSeq 500, CUT&RUN: 553,276
GSM6856399 Isotype_18hpi, rep3 NextSeq 500, CUT&RUN: 772,500
GSM6856400 Isotype_4dpi, rep1 NextSeq 500, CUT&RUN: 3,577,590
GSM6856401 Isotype_4dpi, rep2 NextSeq 500, CUT&RUN: 4,726,550
GSM6856402 Isotype_4dpi, rep3 NextSeq 500, CUT&RUN: 5,433,579

GSM6856403 H3K27ac_18hpi, rep1 NextSeq 500, CUT&RUN: 9,189,271
GSM6856404 H3K27ac_18hpi, rep2 NextSeq 500, CUT&RUN: 7,880,624
GSM6856405 H3K27ac_4dpi, rep1 NextSeq 500, CUT&RUN: 9,666,399
GSM6856406 H3K27ac_4dpi, rep2 NextSeq 500, CUT&RUN: 10,426,321
GSM6856407 H3K4m27me3_18hpi, rep1 NextSeq 500, CUT&RUN: 10,166,831
GSM6856408 H3K4m27me3_18hpi, rep2 NextSeq 500, CUT&RUN: 9,681,981
GSM6856409 H3K4m27me3_4dpi, rep1 NextSeq 500, CUT&RUN: 11,499,242
GSM6856410 H3K4m27me3_4dpi, rep2 NextSeq 500, CUT&RUN: 11,550,515
GSM6856411 H3K4me1_18hpi, rep1 NextSeq 500, CUT&RUN: 9,737,785
GSM6856412 H3K4me1_4dpi, rep1 NextSeq 500, CUT&RUN: 12,460,566
GSM6856413 H3K4me3_18hpi, rep1 NextSeq 500, CUT&RUN: 11,264,721
GSM6856414 H3K4me3_4dpi, rep1 NextSeq 500, CUT&RUN: 10,692,455
GSM6856415 Sox4_18hpi, rep1 NextSeq 2000, CUT&RUN: 31,008,925
GSM6856416 Sox4_18hpi, rep2 NextSeq 2000, CUT&RUN: 16,616,061
GSM6856417 Sox4_18hpi, rep3 NextSeq 2000, CUT&RUN: 31,796,319
GSM6856418 Sox4_4dpi, rep1 NextSeq 2000, CUT&RUN: 29,260,675
GSM6856419 Sox4_4dpi, rep2 NextSeq 2000, CUT&RUN: 46,565,160
GSM6856420 Sox4_4dpi, rep3 NextSeq 2000, CUT&RUN: 110,672,688
GSM6856421 Isotype_18hpi, rep1 NextSeq 2000, CUT&RUN: 4,417,439
GSM6856422 Isotype_18hpi, rep2 NextSeq 2000, CUT&RUN: 5,916,684
GSM6856423 Isotype_18hpi, rep3 NextSeq 2000, CUT&RUN: 7,177,626
GSM6856424 Isotype_4dpi, rep1 NextSeq 2000, CUT&RUN: 34,915,928
GSM6856425 Isotype_4dpi, rep2 NextSeq 2000, CUT&RUN: 36,516,057
GSM6856426 Isotype_4dpi, rep3 NextSeq 2000, CUT&RUN: 52,176
GSM6856427 H3K27ac_18hpi, rep3 NextSeq 2000, CUT&RUN: 9,604,451
GSM6856428 H3K27ac_4dpi, rep3 NextSeq 2000, CUT&RUN: 14,184,246
GSM6856429 H3K4m27me3_18hpi, rep3 NextSeq 2000, CUT&RUN: 11,088,732
GSM6856430 H3K4m27me3_4dpi, rep3 NextSeq 2000, CUT&RUN: 12,909,177
GSM6856431 H3K4me1_18hpi, rep2 NextSeq 2000, CUT&RUN: 13,901,623
GSM6856432 H3K4me1_18hpi, rep3 NextSeq 2000, CUT&RUN: 15,677,903
GSM6856433 H3K4me1_4dpi, rep2 NextSeq 2000, CUT&RUN: 16,681,484
GSM6856434 H3K4me1_4dpi, rep3 NextSeq 2000, CUT&RUN: 14,802,834
GSM6856435 H3K4me3_18hpi, rep2 NextSeq 2000, CUT&RUN: 13,690,804
GSM6856436 H3K4me3_18hpi, rep3 NextSeq 2000, CUT&RUN: 13,471,709
GSM6856437 H3K4me3_4dpi, rep2 NextSeq 2000, CUT&RUN: 17,785,011
GSM6856438 H3K4me3_4dpi, rep3 NextSeq 2000, CUT&RUN: 14,340,054
GSM7656257 BEC_rep1_H3K27ac NextSeq2000, CUT&Tag: 5035661
GSM7656268 BEC_rep1_H3K27me3 NextSeq2000, CUT&Tag: 5,742,230
GSM7656235 BEC_rep1_H3K4me1 NextSeq2000, CUT&Tag: 6,942,655
GSM7656246 BEC_rep1_H3K4me3 NextSeq2000, CUT&Tag: 2,762,276
GSM7656258 BEC_rep2_H3K27ac NextSeq2000, CUT&Tag: 5,227,823
GSM7656269 BEC_rep2_H3K27me3 NextSeq2000, CUT&Tag: 5,853,600
GSM7656236 BEC_rep2_H3K4me1 NextSeq2000, CUT&Tag: 3,272,190
GSM7656247 BEC_rep2_H3K4me3 NextSeq2000, CUT&Tag: 3,041,392
GSM7656248 Hep_rep1_H3K27ac NextSeq2000, CUT&Tag: 6,096,372
GSM7656259 Hep_rep1_H3K27me3 NextSeq2000, CUT&Tag: 4,464,183
GSM7656226 Hep_rep1_H3K4me1 NextSeq2000, CUT&Tag: 4,257,990
GSM7656237 Hep_rep1_H3K4me3 NextSeq2000, CUT&Tag: 2,772,701
GSM7656249 Hep_rep2_H3K27ac NextSeq2000, CUT&Tag: 6,347,670
GSM7656260 Hep_rep2_H3K27me3 NextSeq2000, CUT&Tag: 6,574,466
GSM7656227 Hep_rep2_H3K4me1 NextSeq2000, CUT&Tag: 6,656,272
GSM7656238 Hep_rep2_H3K4me3 NextSeq2000, CUT&Tag: 3,455,839
GSM7656250 Rep_early_rep1_H3K27ac NextSeq2000, CUT&Tag: 5,536,897
GSM7656261 Rep_early_rep1_H3K27me3 NextSeq2000, CUT&Tag: 5,915,382
GSM7656228 Rep_early_rep1_H3K4me1 NextSeq2000, CUT&Tag: 5,081,155
GSM7656239 Rep_early_rep1_H3K4me3 NextSeq2000, CUT&Tag: 3,238,234
GSM7656251 Rep_early_rep2_H3K27ac NextSeq2000, CUT&Tag: 7,659,851
GSM7656262 Rep_early_rep2_H3K27me3 NextSeq2000, CUT&Tag: 7,175,530
GSM7656229 Rep_early_rep2_H3K4me1 NextSeq2000, CUT&Tag: 7,586,464
GSM7656240 Rep_early_rep2_H3K4me3 NextSeq2000, CUT&Tag: 3,930,594
GSM7656252 Rep_intermed_rep1_H3K27ac NextSeq2000, CUT&Tag: 6,998,059
GSM7656263 Rep_intermed_rep1_H3K27me3 NextSeq2000, CUT&Tag: 7,417,902
GSM7656230 Rep_intermed_rep1_H3K4me1 NextSeq2000, CUT&Tag: 6,842,445
GSM7656241 Rep_intermed_rep1_H3K4me3 NextSeq2000, CUT&Tag: 3,117,437
GSM7656253 Rep_intermed_rep2_H3K27ac NextSeq2000, CUT&Tag: 7,206,421
GSM7656264 Rep_intermed_rep2_H3K27me3 NextSeq2000, CUT&Tag: 7,333,679
GSM7656231 Rep_intermed_rep2_H3K4me1 NextSeq2000, CUT&Tag: 2,263,302
GSM7656242 Rep_intermed_rep2_H3K4me3 NextSeq2000, CUT&Tag: 3,962,492

	GSM7656254 Rep_late_rep1_H3K27ac NextSeq2000, CUT&Tag: 3,036,369<br>GSM7656265 Rep_late_rep1_H3K27me3 NextSeq2000, CUT&Tag: 3,003,866<br>GSM7656232 Rep_late_rep1_H3K4me1 NextSeq2000, CUT&Tag: 4,240,138<br>GSM7656243 Rep_late_rep1_H3K4me3 NextSeq2000, CUT&Tag: 889,480<br>GSM7656255 Rep_late_rep2_H3K27ac NextSeq2000, CUT&Tag: 7,503,193<br>GSM7656266 Rep_late_rep2_H3K27me3 NextSeq2000, CUT&Tag: 5,792,009<br>GSM7656233 Rep_late_rep2_H3K4me1 NextSeq2000, CUT&Tag: 6,277,444<br>GSM7656244 Rep_late_rep2_H3K4me3 NextSeq2000, CUT&Tag: 3,699,732<br>GSM7656256 Rep_late_rep3_H3K27ac NextSeq2000, CUT&Tag: 7,169,365<br>GSM7656267 Rep_late_rep3_H3K27me3 NextSeq2000, CUT&Tag: 7,035,887<br>GSM7656234 Rep_late_rep3_H3K4me1 NextSeq2000, CUT&Tag: 5,232,717<br>GSM7656245 Rep_late_rep3_H3K4me3 NextSeq2000, CUT&Tag: 3,437,933
Antibodies	HA-Tag (C29F4) Rabbit mAb Cell Signaling Technology Cat# 3724S<br>Rabbit (DA1E) mAb IgG XP® Isotype Control Cell Signaling Technology Cat# 3900S<br>Anti-Histone H3 (acetyl K27) antibody - ChIP Grade Abcam Cat# ab4729<br>Tri-Methyl-Histone H3 (Lys27) (C36B11) Rabbit mAb Cell Signaling Technology Cat# 9733<br>Anti-Histone H3 (mono methyl K4) antibody - ChIP Grade Abcam Cat# ab8895<br>Anti-Histone H3 (tri methyl K4) antibody - ChIP Grade Abcam Cat# ab8580
Peak calling parameters	Peak calling for histone PTMs was performed using MACS2 without Isotype controls as input files. Narrow peaks for H3K27ac, H3K4me1 and H3K4me3 and broad peaks for H3K27me3 were used with FDR set to 0.01. Called peaks were filtered using the mm10-blacklist.v2 file.<br><br>For Sox4 CUT&RUN samples, which had relatively high background, peak calling was performed using MACS2 with combined Isotype controls of the corresponding time points as input files with FDR set to 0.1. Narrow peaks were used, and the called peaks were filtered using the mm10-blacklist.v2 file.
Data quality	We confirmed acceptable mapping rates and agreement between replicates by Spearman correlation heatmaps and PCA plots as assessed by DiffBind software.
Software	DESeq2 v1.36.0 (Love et al., 2014) https://lashlock.github.io/compbio/R_presentation.html<br>Bowtie2 v2.2.5 (Langmead and Salzberg, 2012) https://bowtie-bio.sourceforge.net/bowtie2/index.shtml<br>Samtools v1.6 (Li, 2011; Li et al., 2009) https://samtools.sourceforge.net/<br>Picard v2.27.4 http://broadinstitute.github.io/picard/<br>Bedtools v2.30.0 https://bedtools.readthedocs.io/en/latest/<br>Macs2 v2.2.7.1 (Feng et al., 2012) https://hbctraining.github.io/Intro-to-ChIPseq/lessons/05_peak_calling_macs.html<br>Deeptools v3.5.1 (Ramírez et al., 2016) https://deeptools.readthedocs.io/en/develop/<br>Homer v4.11 http://homer.ucsd.edu/homer/motif/Tobias v0.13.3<br>DiffBind v3.6.4 (Ross-Innes et al., 2012) https://hbctraining.github.io/Intro-to-ChIPseq/lessons/08_diffbind_differential_peaks.html<br>ChIPseeker v1.32.1 (Yu et al., 2015) https://rdrr.io/bioc/ChIPseeker/f/vignettes/ChIPseeker.Rmd

Flow Cytometry

Plots

Confirm that:

- [x] The axis labels state the marker and fluorochrome used (e.g. CD4-FITC).
- [x] The axis scales are clearly visible. Include numbers along axes only for bottom left plot of group (a 'group' is an analysis of identical markers).
- [x] All plots are contour plots with outliers or pseudocolor plots.
- [x] A numerical value for number of cells or percentage (with statistics) is provided.

Methodology

Sample preparation	For flow cytometry without sorting, whole liver cells were harvested by liver perfusion of liberase enzyme.<br>For FACS sorting, non-parenchymal cells were magnetically eliminated (Dynabeads system) from whole liver cells using antibodies against Cd11b, Cd31 and Cd45. Then, Epcam+ and Epcam- fractions were separated using Epcam microbeads and MACS separation system (Mitenyi).
Instrument	FACS LSR II, FACS Aria II/BD Biosciences
Software	BD FACSDiva8 for data acquisition. Flowjo for visualization and quantification.
Cell population abundance	A small fraction of FACS-sorted cells were analyzed on the sorter, and the population was confirmed to be enriched at 85-100% purity.

Gating strategy	FSC/SSC, doublet exclusion and viability dye used to identify viable single-cell population and exclude debris. Fluorescence minus one (FMO) controls were used to define background.

☒ Tick this box to confirm that a figure exemplifying the gating strategy is provided in the Supplementary Information.
